# Zeta-Carotene Isomerase (Z-ISO) Is Required for Light-Independent Carotenoid Biosynthesis in the Cyanobacterium *Synechocystis* sp. PCC 6803

**DOI:** 10.3390/microorganisms10091730

**Published:** 2022-08-27

**Authors:** Matthew S. Proctor, Felix S. Morey-Burrows, Daniel P. Canniffe, Elizabeth C. Martin, David J. K. Swainsbury, Matthew P. Johnson, C. Neil Hunter, George A. Sutherland, Andrew Hitchcock

**Affiliations:** 1 School of Biosciences, University of Sheffield, Sheffield S10 2TN, UK; 2 Institute of Systems, Molecular & Integrative Biology, University of Liverpool, Liverpool L69 7BE, UK; 3 School of Biological Sciences, University of East Anglia, Norwich NR4 7TJ, UK

**Keywords:** carotenoid, cyanobacteria, *Synechocystis*, zeta-carotene isomerase (Z-ISO), photosynthesis

## Abstract

Carotenoids are crucial photosynthetic pigments utilized for light harvesting, energy transfer, and photoprotection. Although most of the enzymes involved in carotenoid biosynthesis in chlorophototrophs are known, some are yet to be identified or fully characterized in certain organisms. A recently characterized enzyme in oxygenic phototrophs is 15-*cis*-zeta(ζ)-carotene isomerase (Z-ISO), which catalyzes the *cis*-to-*trans* isomerization of the central 15–15′ *cis* double bond in 9,15,9′-tri-*cis*-ζ-carotene to produce 9,9′-di-*cis*-ζ-carotene during the four-step conversion of phytoene to lycopene. Z-ISO is a heme B-containing enzyme best studied in angiosperms. Homologs of Z-ISO are present in organisms that use the multi-enzyme poly-*cis* phytoene desaturation pathway, including algae and cyanobacteria, but appear to be absent in green bacteria. Here we confirm the identity of Z-ISO in the model unicellular cyanobacterium *Synechocystis* sp. PCC 6803 by showing that the protein encoded by the slr1599 open reading frame has ζ-carotene isomerase activity when produced in *Escherichia coli*. A *Synechocystis* Δslr1599 mutant synthesizes a normal quota of carotenoids when grown under illumination, where the photolabile 15–15′ *cis* double bond of 9,15,9′-tri-*cis*-ζ-carotene is isomerized by light, but accumulates this intermediate and fails to produce ‘mature’ carotenoid species during light-activated heterotrophic growth, demonstrating the requirement of Z-ISO for carotenoid biosynthesis during periods of darkness. In the absence of a structure of Z-ISO, we analyze AlphaFold models of the *Synechocystis*, *Zea mays* (maize), and *Arabidopsis thaliana* enzymes, identifying putative protein ligands for the heme B cofactor and the substrate-binding site.

## 1. Introduction

**Figure 1:**
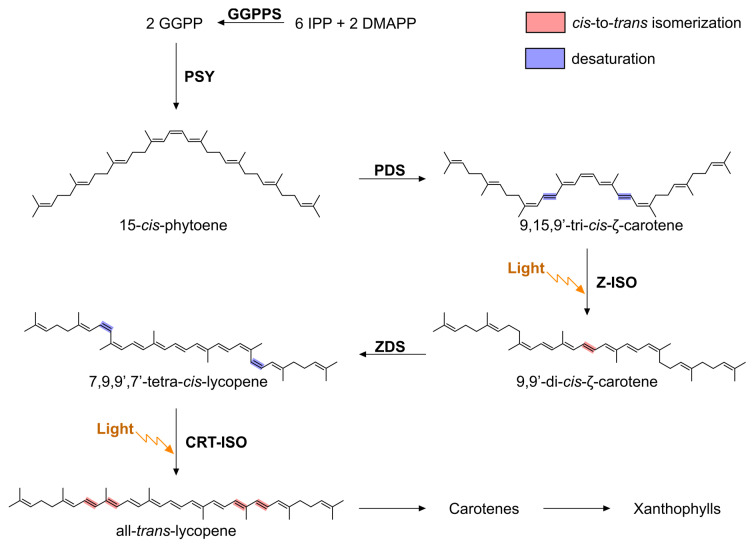
Overview of carotenoid biosynthesis in oxygenic phototrophs focusing on the steps in the conversion of phytoene to lycopene. The products of the MEP pathway, IPP and DMAPP, are condensed to form GGPP by the enzyme GGPP synthase (GGPPS). Two molecules of GGPP are subsequently condensed by the enzyme phytoene synthase (PSY), producing 15-*cis*-phytoene. Phytoene desaturase (PDS) and ζ-carotene desaturase (ZDS) introduce four double bounds by desaturation of the carotenoid backbone (blue highlight) to produce all-*trans*-lycopene, with *cis*-to-*trans* isomerization of bonds in the intermediate species (red highlight) carried out by Z-ISO and CRT-ISO; these *cis*-bonds are photo-labile and can be isomerized non-enzymatically by light (indicated by orange arrows). Subsequent modification of all-*trans*-lycopene (enzymatic steps not shown) yields the carotene and xanthophyll species used for light harvesting, photoprotection, and structural stabilization of proteins and lipid membranes.

Carotenoids are pigments with important structural, light-harvesting, and photoprotective roles in photosynthetic pigment-protein complexes and membranes [1,2]. Carotenoid biosynthesis in phototrophs (for reviews see [3,4]) begins with the condensation of the products of the 2-C-methyl-D-erythritol 4-phosphate (MEP) pathway: isopentyl diphosphate (IPP) and dimethylallyl diphosphate (DMAPP). This generates geranyl pyrophosphate (GPP), which is converted to geranylgeranyl pyrophosphate (GGPP) (via farnesyl-pyrophosphate) by condensation with two further IPP molecules, prior to the production of 15-*cis*-phytoene (phytoene) by the condensation of two molecules of GGPP. How phytoene is converted to carotenoids with longer conjugation length (*N*) polyene chains differs between phototrophic organisms. In phototrophic bacteria, phytoene desaturase (CrtI) catalyzes sequential desaturations of phytoene (colorless with three conjugated carbon–carbon double bonds; *N* = 3) to produce neurosporene (yellow, three desaturations of phytoene; *N* = 9) or lycopene (orange/red; four desaturations of phytoene; *N* = 11) [3,4]. Neurosporene and/or lycopene are subsequently modified by additional enzymes to produce the carotenoids that are utilized for light harvesting and photoprotection. Conversely, oxygenic phototrophs (cyanobacteria, algae, and plants), green sulfur bacteria (GSB), and Chloroacidobacteria use a multi-enzyme pathway to synthesize lycopene from phytoene (Figure 1); the four enzymes are phytoene desaturase (PDS/CrtP; catalyzes two desaturations of lycopene), zeta(ζ)-carotene isomerase (Z-ISO), ζ-carotene desaturase (ZDS/CrtQ; catalyzes two desaturations of ζ-carotene), and the prolycopene isomerase (CRT-ISO/CrtH) [3,4].

Z-ISO is the most recently identified early carotenoid biosynthesis enzyme; the *Zea mays* (maize) enzyme has been demonstrated to be a heme B-containing integral membrane protein that catalyzes the redox-regulated *cis*-to-*trans* conversion of the 15-*cis* double bond in 9,15,9′-tri-*cis*-ζ-carotene to form 9,9′-di-*cis*-ζ-carotene [5,6]. Z-ISO homologs are present in plants, algae, diatoms, and cyanobacteria; the enzymes from maize and *Arabidopsis thaliana* (hereafter *Arabidopsis*) are the best studied [5,7,8] but Z-ISO has also been experimentally identified in *Oryza sativa* (rice) [9,10], the filamentous cyanobacterium *Arthospira* [11], the eukaryotic microalga Euglena [7,11], and the fruits *Citrus sinensis* [12] and tomato [13]. Interestingly, GSB and species of the *Chloracidobacterium* genus appear to lack Z-ISO [8,11]. Maize and *Arabidopsis* mutants accumulate 9,15,9′-tri-*cis*-ζ-carotene and lack carotenoids in the dark, as well as having delayed greening and a lower carotenoid content when exposed to light [8]. In rice, Z-ISO links carotenoid and plant hormone biosynthesis to regulate/modulate photosynthesis and tillering [9,10]. The silencing of *Z-ISO* mRNA in a tomato plant results in a reduction in lycopene and an increase in phytoene, phytofluene, and ζ-carotene [14] and a non-functional splice variant of Z-ISO is associated with the yellow pigmentation of the “Pinalate” sweet orange mutant, which accumulates unusual proportions of 9,15,9′-tri-*cis*- and 9,9′-di-*cis*-ζ-carotene, a phenotype partially restored by exposure to high light [12].

Carotenoid biosynthesis has been extensively characterized in model cyanobacteria such as *Synechocystis* sp. PCC 6803 (hereafter *Synechocystis*), where carotenoids play important functional and structural roles in photosystems (PS) I and II [15,16], and are also found in the cytochrome *b*_6_*f* complex [17,18] and the NADH dehydrogenase-like photosynthetic complex I [19,20]. *Synechocystis* mutants lacking *pds* (*crtP*, slr1254) or *zds* (*crtQ*, slr0940) accumulate phytoene or ζ-carotene, respectively, display very slow glucose-dependent growth, are acutely light sensitive, and lack PSII [21]. Conversely, the deletion of sll0033 (*crtH*), which encodes the CRT-ISO enzyme that converts 7,9,7’,9′-tetra-*cis*-lycopene (prolycopene) to all-*trans*-lycopene, results in a strain that can produce mature carotenoids in the light, albeit in different ratios to the WT organism, due to the photo-isomerization of prolycopene [22,23,24]. However, the Δ*crtH* strain predominantly accumulates *cis*-isomers of carotenes and does not make β-carotene under light-activated heterotrophic growth (LAHG) conditions (darkness with a short pulse of light every 24 h), and therefore it cannot assemble PSII [25].

CRT-ISO isomerizes the adjacent 7,9 and 7′9′ *cis*-pairs of double bonds in 7,9,7’,9′-tetra-*cis*-lycopene but does not act on the 15,15′ *cis* double bond of 9,15,9′-tri-*cis*-ζ-carotene [26]. This 15-*cis* double bond is also photo-labile and can be photo-isomerized, but this appears to be less efficient than the enzyme-catalyzed reaction [7,8]. As Z-ISO is essential for carotenogenesis in dark plant tissues such as the roots, etiolated leaves, and endosperm [7], we predicted that the same would be true for carotenoid biosynthesis in cyanobacteria during periods of darkness. Here, we verify the ζ-carotene isomerase activity of the *Synechocystis* Z-ISO homolog (encoded by the slr1599 locus) by heterologous production of Slr1599 in a 9,15,9′-tri-*cis*-ζ-carotene-accumulating strain of *Escherichia coli*. We also show that Z-ISO is needed for carotenoid biosynthesis in the absence of light in a *Synechocystis* Δslr1599 mutant, which to our knowledge is the first characterization of a cyanobacterial *Z-ISO* deletion strain. Despite repeated attempts, we were unable to purify the *Synechocystis* Z-ISO for spectral analysis and structure determination, but a comparison of AlphaFold structural models of the *Synechocystis* and plant enzymes shows conservation of putative heme-ligating residues and allows us to predict the 9,15,9′-tri-*cis*-ζ-carotene-binding site.

## 2. Materials and Methods

### 2.1. Generation of Constructs to Test Synechocystis Z-ISO Activity in Escherichia coli

To test the isomerase activity of Slr1599 in *E. coli*, the slr1599 gene was PCR-amplified from the *Synechocystis* genome and cloned into the NdeI and XhoI sites of MCS2 of pCDFDuet^Tm^-1 (Novagen). The resulting sequence-verified construct (pAH592) was introduced into *E. coli* BL21(DE3) along with the pAC-ZETAipi plasmid [27] (purchased from Addgene, plasmid #53284) with selection on LB agar containing streptomycin (50 µg mL^−1^) and chloramphenicol (34 µg mL^−1^). pCDFDuet^Tm^-1 constructs containing the maize *Z-ISO* gene (synthesized as a gBLOCK by Integrated DNA Technologies) at MCS2 (NdeI/XhoI sites; pAH595), *Synechocystis zds* (*crtQ*; slr0940) at MCS1 (NcoI/SalI sites; pAH585), *zds* at MCS1 and slr1599 at MCS2 (pAH587), and *zds* at MCS1 and the maize *Z-ISO* gene at MSC2 (pAH590) were also generated. Further details of plasmids and primers used in this study are provided in Supplementary Table S1 and Table S2, respectively. PCRs were performed using Q5^®^ Hot Start High-Fidelity 2x Master Mix (New England Biolabs (UK) Ltd.) and restriction digests using Thermo Scientific™ FastDigest enzymes. Ligation reactions were conducted using T4 DNA ligase (New England Biolabs (UK) Ltd.) and all cloning steps used *E. coli* JM109 competent cells (Promega). DNA was purified using the FastGene Gel/PCR Extraction Kit or Plasmid Mini Kit (both from Nippon Genetics), as appropriate, and automated Sanger sequencing was performed by Eurofins.

### 2.2. Carotenoid Analysis by Reverse-Phase High-Performance Liquid Chromatography (RP-HPLC)

For production of carotenoids in *E. coli*, 300 µL of a 5 mL overnight culture was used to inoculate 15 mL auto-induction Terrific Broth (TB) base without trace elements (Formedium) with relevant antibiotics in 50 mL opaque falcon tubes. These cultures were incubated in darkness at 37 °C for 24 h in a rack set at a 45° angle on a shaking platform rotating at 220 rpm. All subsequent steps were performed in the dark. Cells were harvested by centrifugation at 4600× *g* for 10 min at 4 °C, washed with ice-cold 30 mM HEPES pH 7.4, and transferred to 2 mL screw-capped tubes. Pigments were extracted from cell pellets by adding 700 µL of 7:2 acetone:methanol (*v*/*v*), vortex-mixing for 30 s, and incubating on ice for 20 min in the dark. The extracts were clarified by centrifugation (15,000× *g* for 5 min at 4 °C), supernatants were transferred to fresh tubes to which 30 µL 5 M NaCl and 700 µL hexane were added, mixed, and allowed to partition. The upper hexane phase was transferred to a 2 mL glass vial and evaporated to dryness in a vacuum centrifuge at ambient temperature. Dried pigments were dissolved in 150 µL acetonitrile, and immediately analyzed on an Agilent 1200 HPLC system. ζ-carotene species were separated on a YMC30 C30 RP column (3 μm particle size; 250 mm × 4.6 mm). An isocratic buffer composed of 40:30:30 acetonitrile:methanol:tetrahydrofuran (*v*/*v*/*v*) was used to separate carotenoids at 1 mL min^−1^ at 40 °C, and elution of pigments was monitored by absorbance at 425 nm. Lycopene species were separated on a Discovery HS C18 column (5 μm particle size; 250 mm × 4.6 mm) according to the slightly modified method described in our previous work [28]. 

*Synechocystis* carotenoids were extracted from cell pellets washed with ice-cold 30 mM HEPES pH 7.4 by vortex mixing in 100% methanol for 30 s, the extracts were clarified by centrifugation (as above), and the supernatants were analyzed following the same protocol used for analysis of lycopene species in *E. coli*.

### 2.3. Generation and Growth of Synechocystis Strains

*Synechocystis* strains were grown photoautotrophically under ~50 µmol photons m^−2^ s^−1^ constant illumination at 30 °C in sterile BG11 medium [29] buffered with 10 mM TES (N-Tris(hydroxymethyl)methyl-2-aminoethanesulfonic acid)-KOH pH 8.2 (BG11-TES). For LAHG, cultures were grown in BG11-TES supplemented with 5 mM glucose in complete darkness apart from a 5-min pulse of white light (~40 µmol photons m^−2^ s^−1^) once every 24 h [30]. For growth on solid media, 1.5% (*w*/*v*) bactoagar and 0.3% (*w*/*v*) sodium thiosulphate were added to BG11-TES. Antibiotics were included where appropriate, as detailed below. For attempted purification of FLAG-tagged Slr1599, cultures were grown either photoautotrophically (~50–100 µmol photons m^−2^ s^−1^) or under LAHG conditions (as above) in 8 L vessels mixed by bubbling with sterile air and maintained at 30 °C by a temperature coil connected to a thermostat-controlled circulating water bath.

To make a Δslr1599 deletion mutant, a linear mutagenesis construct was generated from three individual PCR product templates by overlap-extension (OLE)-PCR. The first fragment consisted of 427 bp upstream of the slr1599 start codon (see Section 3.1), the first 80 bp of the slr1599 gene, and 26 bp of homology to the start of the erythromycin-resistance cassette from pERY [31] and was amplified with primers AH912 and AH913. The second fragment consisted of the erythromycin resistance gene, which was amplified from pERY using primers AH595 and AH596. The third fragment consisted of 26 bp of homology to the end of the erythromycin resistance cassette, the final 80 bp of slr1599, and 391 bp downstream of the slr1599 stop codon and was amplified with primers AH914 and AH915. The purified fragments were mixed in equimolar amounts and joined by OLE-PCR using primers AH912 and AH915, and the resulting construct was sequenced to confirm correct assembly (Eurofins). Following introduction of the OLE-PCR product into wild-type (WT) *Synechocystis* by natural transformation, transformants were selected on BG11 agar containing 5 µg mL^−1^ erythromycin and single colonies were picked and streaked onto BG11 agar containing a progressively doubled concentration of erythromycin up to 40 µg mL^−1^. Full segregation of the Δslr1599 mutant was confirmed by PCR amplification with primers AH912 and AH915 and Sanger sequencing of the resultant purified PCR product (Eurofins).

Complementation of Δslr1599 was performed by integrating slr1599 or the *Arabidopsis Z-ISO* gene at the *psbAII* locus using the pPD-*N*FLAG plasmid [32,33]. slr1599 was amplified from *Synechocystis* WT genomic DNA using the primers AH955 and AH956 and cloned into the NotI and BglII sites of pPD-*N*FLAG downstream of the sequence encoding an N-terminal 3×FLAG tag. Attempts to insert slr1599 into the pPD-*C*FLAG plasmid [34] —such that when integrated in place of *psbAII* in the *Synechocystis* genome the construct would encode Z-ISO with a C-terminal 3×FLAG tag—were unsuccessful, presumably due to toxicity of this fusion protein in the *E. coli* JM109 cells used for cloning. Therefore, we PCR-amplified a linear DNA fragment consisting of slr1599 followed by the sequence adding the C-terminal FLAG-tag and the kanamycin resistance gene and flanked by the *psbAII* homology arms from the ligation reaction using primers AH198 and AH199, which was verified by sequencing the PCR product. The *Arabidopsis Z-ISO* gene (At1g10830) was synthesized (Integrated DNA Technologies) lacking bases 4–174 (the sequence encoding the N-terminal chloroplast transit peptide) and with the sequence encoding a Ser-Ala linker followed by the Strep-tag^®^ II added to the 3′ end of the gene, and then cloned into the NdeI and BglII sites of pPD-*N*FLAG. The sequence-verified plasmids (pAH445 and pAH436; Table S1) and linear PCR fragment were individually transformed into the Δslr1599 mutant and, following selection on BG11 agar containing 7.5 µg mL^−1^ kanamycin, colonies were picked and segregated by streaking onto increasing concentrations of kanamycin up to 30 µg mL^−1^. Full segregation of the *psbAII* locus was determined by PCR with primers AH47 and AH48, and automated Sanger sequencing (Eurofins) confirmed the correct insertion of slr1599 or At1g10830. It was not possible to isolate kanamycin-resistant colonies of Δslr1599 for the C-terminally FLAG-tagged *Synechocystis* enzyme.

*Synechocystis* slr1599 point mutants were generated with the QuikChange II Site-Directed Mutagenesis Kit (Agilent Technologies), according to the manufacturer’s instructions, using pAH445 as template and the primer pairs detailed in Table S2. The resulting plasmids were sequence-verified prior to introduction to the *Synechocystis* Δslr1599 mutant and after PCR amplification of the segregated *psbAII* locus from *Synechocystis* strains (as above).

### 2.4. Purification of Recombinant Maize Z-ISO

Maize Z-ISO fused to maltose-binding protein (MBP) was purified following a protocol adapted from those described by Beltrán et al [5,35,36]. The sequence of the codon-optimized construct that was cloned into pET21a(+), yielding plasmid pAH545, is provided in Table S3. Twelve 500 mL cultures of *E. coli* BL21(DE3) harboring pAH545 were grown in 2xYT medium (10 g L^−1^ yeast extract, 16 g L^−1^ tryptone, and 5 g L^−1^ NaCl) containing 100 µg mL^−1^ ampicillin at 37 °C with shaking (200 rpm) to an optical density at 600 nm of 0.6, at which point 1 mM isopropyl β-d-1-thiogalactopyranoside was added to each culture and the temperature was lowered to 28 °C for overnight (~18 h) incubation. Cells were collected by centrifugation at 8671× *g* for 10 min at 4 °C and resuspended in 50 mM Tris-HCl pH 7.6, 300 mM NaCl, 5% (*w*/*v*) glycerol, 0.5 mM dithiothreitol (buffer A) supplemented with a Roche protease inhibitor tablet, and a small spatula each of DNase I and lysozyme. The cell suspension was cooled on ice and lysed by two passes through a chilled French press at 18,000 psi with 5 min of chilling on ice between cycles. Lysed cells were centrifuged at 7025× *g* for 20 min at 4 °C to remove cell debris and unbroken cells, and the supernatant was centrifuged again at 48,400× *g* for 30 min at 4 °C to pellet *E. coli* membranes. The pellet (membrane fraction) was resuspended in 80 mL buffer A and n-dodecyl-β-D-maltoside (β-DDM; Anatrace) was added to a final concentration of 1.5% (*w*/*v*) before a 1-h period of incubation in the dark at 4 °C with gentle agitation. The solubilized membrane solution was centrifuged at 48,400× *g* for 30 min at 4 °C and the supernatant was retained, diluted 2x in buffer A, and incubated with 3 mL of pre-washed amylose resin (New England Biolabs (UK) Ltd.) overnight at 4 °C with gentle agitation. The next day, the resin was collected by filtration through a 0.22 µm spin column, washed with 20 resin volumes of buffer A containing 0.04% (*w*/*v*) β-DDM, and bound protein was eluted in the same buffer supplemented with 10 mM maltose. Eluted protein was directly analyzed by absorbance spectroscopy in a UV–Vis cuvette (Sarstedt Inc.) using a Cary 60 UV-Vis spectrophotometer (Agilent Technologies). For redox difference spectra, the protein was oxidized by incubation with a few grains of potassium ferricyanide and then reduced by addition of a small quantity of sodium dithionite. Spectra were measured following 1 min of incubation with each reagent.

Point mutants in the maize *Z-ISO* gene were generated with the Quikchange II Site-Directed Mutagenesis Kit (Agilent Technologies) using pAH545 as template and the primer pairs detailed in Table S2. The variant proteins were purified in the same way as WT maize Z-ISO.

### 2.5. Attempted Purification of Recombinant and Native Slr1599

We attempted to purify Slr1599 as an MBP-fusion protein using the same growth conditions and purification procedures as for the maize enzyme (as described in Section 2.4). We initially generated a construct (pAH546) in which the maize *Z-ISO* gene in pAH545 was replaced with a codon-optimized (for *E. coli*) slr1599 gene (see Table S1 for plasmid details and Table S3 for the full annotated gene sequence). A second construct (pAH565, Table S1) in which the sequence encoding the single transmembrane helix (TMH) protein YnhF was fused to the 5′ end of slr1599 was also generated (see Table S3 for sequence). Constructs in which slr1599 was inserted into the commercial pMAL-c5X (pAH424) and pMAL-p5X (pAH707) plasmids (both New England Biolabs (UK) Ltd.) were also generated (see Tables S1–S3 for details).

Attempted immunoprecipitation of FLAG-tagged Slr1599 from β-DDM-solubilized membranes isolated from the Δslr1599 FLAG-Slr1599 strain of *Synechocystis* was performed as reported in our previous work [28,34].

### 2.6. Computational Modelling and Ligand Docking

AlphaFold models for the maize and *Arabidopsis* Z-ISO enzymes were downloaded from UniProt (https://www.uniprot.org/; peptide sequence identities B4FHU1 (AF-B4FHU1-F1) and Q9SAC0 (AF-Q9SAC0-F1), respectively (accessed on 1 June 2022). The UniProt entry for Slr1599 is N-terminally truncated (see Section 3.1) so the model for *Synechocystis* Z-ISO was generated using AlphaFold v2.1.2 [37] (git commit e93a9eb) with full database searching and the monomer model parameter, which was run on a Dell PowerEdge C4130, housed within the University of Sheffield SHARC high-performance computing cluster, and equipped with an Intel Xenon E5-2630 v3 CPU, 64 GB DDR4 ECC-RAM, and eight NVIDIA Tesla K80 GPUs. Mutated Z-ISO structures were generated in ChimeraX [38] using the AlphaFold structures and scored using the Rosetta membrane protein-scoring suite [39]. Mutants were chosen based on previous biochemical studies [5,6] and rational interrogation of the AlphaFold structures.

The AlphaFold structures of the *Arabidopsis* and maize Z-ISO enzymes were used to dock heme B, and these heme-bound structures were subsequently used for docking of 9,15,9′-tri-*cis*-ζ-carotene. The 3D ligand structure of heme B (HEM-model spatial data file (SDF)) was downloaded from RCSB PDB (https://www.rcsb.org/). We could not find the 3D structure of 9,15,9′-tri-*cis*-ζ-carotene in any database and so generated one from the 2D SDF using ChimeraX, predicting bond lengths by bond order. AutoDock structure files containing structure, partial charges, and rotational freedoms (.pdbqt files) were generated with AutoDock Tools [40]. Rotational freedom was removed from any bonds involved in the 9,15,9′-tri-*cis*-ζ-carotene π-conjugation system. Heme B was docked into both structures by searching a 125,000 Å^3^ volume, investigating 10,000 different binding modes. Using the *Arabidopsis* model, two binding sites were identified with estimated ~9 kcal mol^−1^ binding energy; however, only one was near prototypical bis-His ligands predicted by the AlphaFold structures. This informed a more targeted docking of heme B into the maize Z-ISO structure, where 1000 binding modes in a 2744 Å^3^ volume around the putative bis-His heme-binding site were investigated. The lowest-energy heme B-binding mode was used to generate a new maize Z-ISO-heme B PDB structure, which was used to dock 9,15,9′-tri-*cis*-ζ-carotene, searching 10,000 binding modes in a 125,000 Å^3^ volume.

## 3. Results and Discussion

### 3.1. Identification of a Z-ISO Homolog in Synechocystis

To our knowledge, at the time of writing, the experimental verification of a cyanobacterial Z-ISO has only been reported for the enzyme from the filamentous, non-N_2_-fixing cyanobacterium *Arthrospira platensis* NIES-39, where activity was demonstrated by heterologous production in an *E. coli* strain that accumulated 9,15,9′-tri-*cis*-ζ-carotene in darkness [11]. The same authors also identified Z-ISO homologs in other *Arthrospira* species, the model cyanobacteria *Synechocystis* and *Synechococcus* sp. PCC 7002 (hereafter *Synechococcus* 7002), and *Prochlorococcus marinus* str. SS35 [11]. Zhu et al [41] had earlier identified the same 238 amino acid protein (SYNPCC7002_A1197) as the likely ζ-carotene isomerase in *Synechococcus* 7002, and the locus predicted to encode Z-ISO in the original genome-sequenced *Synechocystis* Kazusa substrain [42] is slr1599 [43].

**Figure 2:**
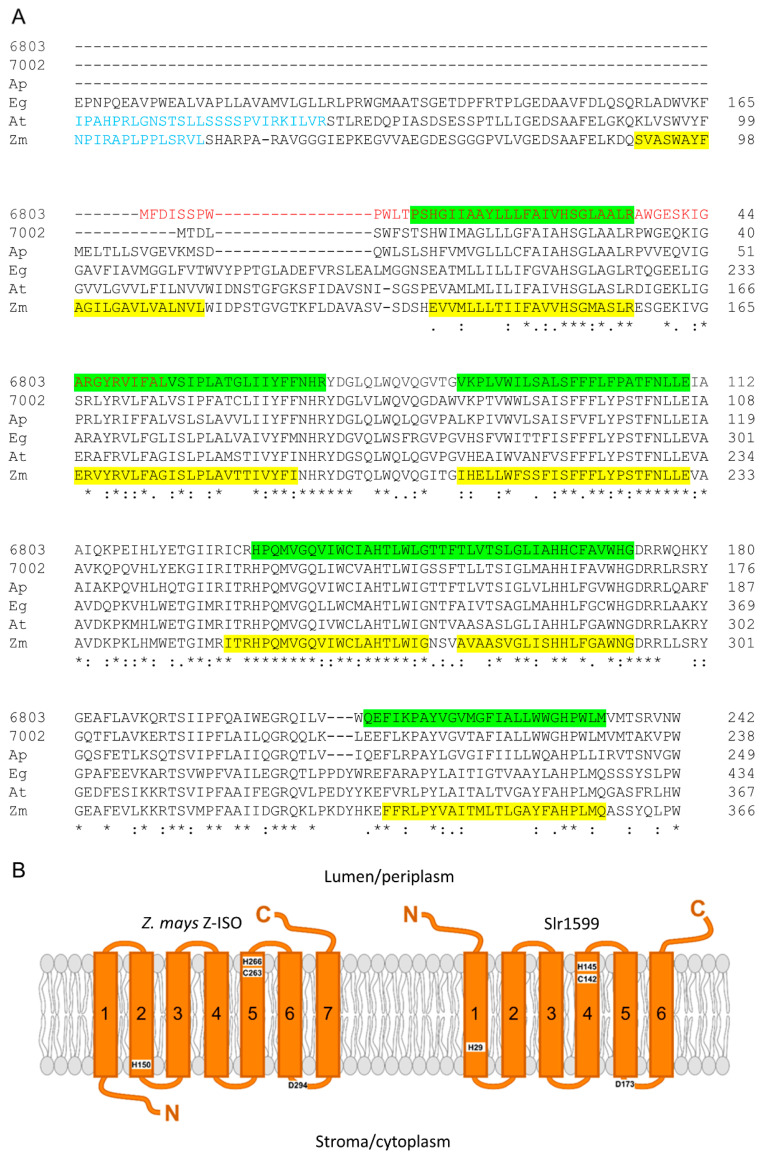
Sequence alignment of Slr1599 with known Z-ISO enzymes from other oxygenic phototrophs. (**A**) Sequence alignment of Slr1599 (6803) with Z-ISO homologs from *Synechococcus* 7002 (7002), *A. platensis* (Ap), *E. gracilis* (Eg), *A. thaliana* (At), and *Z. mays* (Zm). The N-terminal sequence in Slr1599 that is ‘missing’ in some databases is indicated in red font. Note that the eukaryotic enzymes (Eg, At and Zm) are N-terminally truncated and, where appropriate, the sequence corresponding to their chloroplast transit peptides is shown in blue. The position of predicted TMHs in Slr1599 (six; note that helices 4 and 5 are consecutive) and the *Z. mays* (seven) enzyme are highlighted in green and yellow, respectively. Residue numbers are shown on the left and the degree of conservation is shown below the sequence alignment, where ‘*’ indicates the residue is identical, ‘:’ = a conservative substitution, and ‘.’ = a semi-conservative substitution. (**B**) Schematic showing the predicted number of TMHs for Slr1599 in comparison to the *Z. mays* Z-ISO. One fewer TMH in the *Synechocystis* enzyme means that the N-terminus is predicted to be localized to the thylakoid lumen, in contrast to the plant Z-ISO, where the extra TMH leads to a predicted location for the N-terminus on the stromal side of the membrane. Four residues previously implicated as having potential structural/functional roles in the maize Z-ISO enzyme [5,6], and investigated here in Slr1599 (Section 3.4), are indicated on the schematics.

In the UniProt (https://www.uniprot.org/uniprot/P72984), KEGG (https://www.genome.jp/dbget-bin/www_bget?syn:slr1599), and CYORF (http://cyano.genome.jp/cgi-bin/cyorf_view.cgi?ORG=syn&ACCESSION=slr1599) databases (accessed on 1 June 2022), slr1599 is predicted to encode a 188 amino acid protein, ~50 residues smaller than other cyanobacterial Z-ISO homologs [11,41]. This is due to an apparent misidentification of the start codon; the translation of the DNA immediately upstream of slr1599 in combination with analysis of other *Synechocystis* substrains (where the protein is predicted to range from 229–247 amino acids) and an alignment with known Z-ISO enzymes from other organisms indicates that the gene likely starts 162 bp upstream of the annotated GTG start codon, encoding a 242 amino acid protein (Figure 2A). Like other Z-ISO enzymes, Slr1599 is annotated as an NnrU domain-containing protein [8], and similar to other cyanobacterial Z-ISO homologs it is predicted to contain six TMHs (Figure 2B), compared to seven for the plant enzymes [5,12].

### 3.2. Slr1599 Displays Z-ISO Activity in Escherichia coli

**Figure 3:**
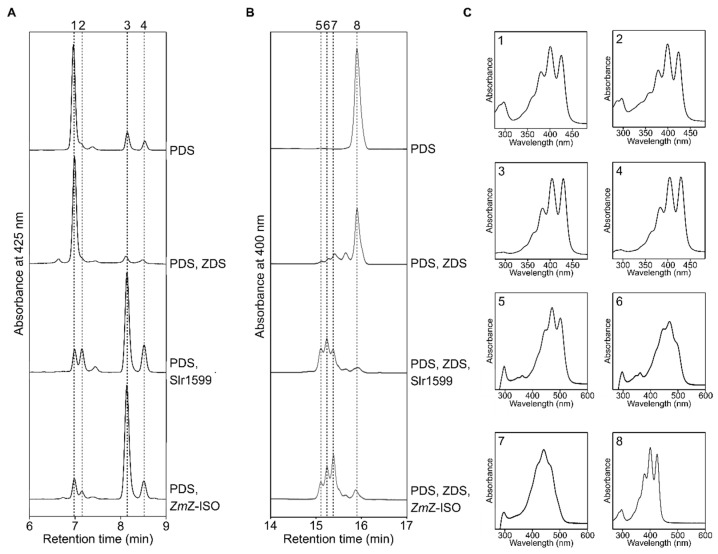
Slr1599 isomerizes 9,15,9′-tri-*cis*-ζ-carotene when produced in *E. coli*. (**A**) RP-HPLC analysis of carotenoids extracted from dark-grown *E. coli* cells containing pAC-ZETAipi and either empty pCDFDuet^Tm^-1 (labeled PDS), pCDFDuet^Tm^-1 containing *Synechocystis crtQ* (PDS, ZDS), pCDFDuet^Tm^-1 containing *Synechocystis* slr1599 (PDS, Slr1599), or pCDFDuet^Tm^-1 containing the maize *Z-ISO* gene (PDS, *Zm*Z-ISO). Eluted pigments were detected by monitoring absorbance at 425 nm. (**B**) RP-HPLC profile of carotenoids extracted from dark-grown *E. coli* cells harboring empty pCDFDuet^Tm^-1 (PDS), pCDFDuet^Tm^-1 containing *Synechocystis crtQ* (PDS, ZDS), pAC-ZETAipi and pCDFDuet^Tm^-1 containing both *crtQ* and slr1599 (PDS, ZDS, Slr1599) or pAC-ZETAipi and pCDFDuet^Tm^-1 containing both *crtQ* and the maize Z-ISO gene (PDS, ZDS, *Zm*Z-ISO). Eluted pigments were detected by monitoring absorbance at 400 nm. (**C**) Absorbance spectra of the numbered carotenoid species in panels (A) and (B). Peaks 1 and 8 = 9,15,9′-tri-*cis*-ζ-carotene; peak 2 = alternative central *cis*-isomer of ζ-carotene [44]; peak 3 = 9,9′-di-*cis*-ζ-carotene; peak 4 = all-*trans* ζ-carotene [44]; peaks 5–7 = *cis*-lycopene isomers [23].

To test the function of Slr1599, we expressed slr1599 in an *E. coli* strain containing pAC-ZETAipi, which contains the *Pantoea agglomerans* (previous known as *Erwinia herbicola*) *crtE* (encodes GGPPS), *crtB* (encodes PSY), and *idi* (encodes IPP isomerase, which interconverts IPP and DMAPP) genes and the *Synechococcus* sp. PCC 7942 *crtP* gene (encodes PDS) to drive the production of the substrate of Z-ISO, 9,15,9′-tri-*cis*-ζ-carotene [27]. In agreement with the previously reported data, *E. coli* cells containing pAC-ZETAipi and empty pCDFDuet^Tm^-1 accumulated primarily 9,15,9′-tri-*cis*-ζ-carotene (Figure 3A, peak 1) when grown in darkness, with small additional peaks that we assigned as 9,9′-di-*cis*-ζ-carotene (peak 3) and all-*trans*-ζ-carotene (peak 4) based on previous analyses [11,44]. The same was true for pAC-ZETAipi plus pCDFDuet^Tm^-1 with the *Synechocystis zds* (*crtQ*) gene, consistent with the fact that ZDS cannot act on 9,15,9′-tri-*cis*-ζ-carotene [26].

Upon the co-transformation of *E. coli* with pAC-ZETAipi and pCDFDuet^Tm^-1 carrying either maize *Z-ISO* or slr1599, the size of the 9,15,9′-tri-*cis*-ζ-carotene peak (no. 1) was significantly decreased and that of the 9,9′-di-*cis*-ζ-carotene peak (no. 3) increased correspondingly (Figure 3A), showing that both enzymes can isomerize the 15–15′ *cis* double bond of 9,15,9′-tri-*cis*-ζ-carotene. Furthermore, when *zds* and slr1599 or *zds* and the maize *Z-ISO* gene were present in the pCDFDuet^Tm^-1, ζ-carotene species were almost completely absent and a variety of new carotenoid species (Figure 3B, peaks 5–7), likely *cis*-isomers of lycopene [23], were produced; these *cis*-lycopene species were absent in the corresponding samples lacking Z-ISO, which accumulated 9,15,9′-tri-*cis*-ζ-carotene (peak 8, Figure 3B), as in the absence of *zds*. Taken together, these data demonstrate that slr1599 encodes an enzyme with 9,15,9′-tri-*cis*-ζ-carotene isomerase activity akin to previously characterized Z-ISO enzymes [5,7,8,11].

### 3.3. The Synechocystis Z-ISO Is Required for Carotenoid Biosynthesis in Darkness In Vivo

**Figure 4:**
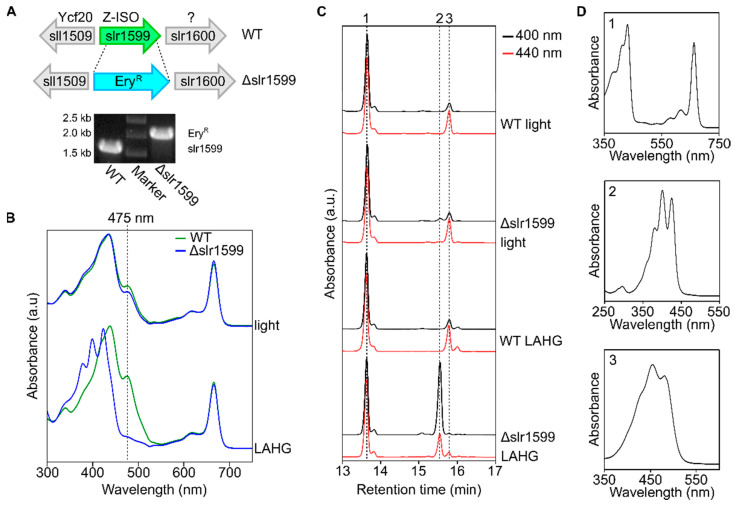
Carotenoid composition of WT and Δslr1599 strains of *Synechocystis* grown under photoautotrophic and LAHG conditions. (**A**) Schematic showing generation of the Δslr1599 *Synechocystis* mutant by partial replacement of slr1599 (green) with the erythromycin resistance gene (Ery^R^) by homologous recombination (indicated by the dotted lines). Segregation of the mutant was confirmed by PCR amplification of the slr1599 locus in comparison to the isogenic WT parent strain. (**B**) Absorbance spectra of methanolic extracts of the WT (green trace) and Δslr1599 (blue trace) strains grown under constant illumination (light) or LAHG conditions. The black dotted line highlights the contribution of ‘mature’ carotenoids to the WT spectra at ~475 nm, which is largely depleted in the mutant under LAHG conditions. (**C**) RP-HPLC analysis of pigments extracted from WT and Δslr1599 cells grown under photoautotrophic (light) or LAHG conditions. Chlorophyll (peak 1), 9,15,9′-tri-*cis*-ζ-carotene (peak 2) and β-carotene (peak 3) were detected by monitoring absorbance at 400 nm (black traces) and 440 nm (red traces); traces are normalized to the height of the Chl peak. (**D**) Absorbance spectra of the numbered pigment species in panel (**C**).

We next examined the role of Slr1599 in *Synechocystis* carotenoid biosynthesis in vivo. A mutant lacking slr1599 was generated by the partial replacement of the open reading frame with an erythromycin resistant cassette (Figure 4A). Under photoautotrophic conditions (constant illumination), the Δslr1599 mutant grew similarly to the WT, and the absorbance spectra of both strains were almost identical (Figure 4B). The production of β-carotene was used to indicate Z-ISO activity; β-carotene levels were similar in the WT and Δslr1599 strains grown under constant illumination, indicating that the non-enzymatic photo-isomerization of the 15,15′ *cis* double bond of 9,15,9′-tri-*cis*-ζ-carotene can occur in the absence of Slr1599 (Figure 4C). This is analogous to the case with the *Synechocystis* Δ*crtH* mutant, where the loss of CRT-ISO does not result in a significant carotenoid defect in the light due to the photolability of the *cis* double bonds of 7,9,7’,9′-tetra-*cis*-lycopene, such that all-*trans*-lycopene is formed non-enzymatically [23].

*Synechocystis* cannot grow in complete darkness even when supplemented with glucose due to its requirement for at least a short (~5 min) daily pulse of light [30]. The pulse of light that permits LAHG is not sufficient to photo-isomerize the *cis*-bonds in 7,9,7’,9′-tetra-*cis*-lycopene and so a Δ*crtH* mutant cannot produce carotenoids under these conditions [23]. We tested the Δslr1599 mutant under the same LAHG conditions and observed an almost complete loss of β-carotene accompanied by a significant accumulation of ζ-carotene (Figure 4C), showing that unlike the WT, the mutant is unable to convert 9,15,9′-tri-*cis*-ζ-carotene to 9,9′-di-*cis*-ζ-carotene in the absence of prolonged illumination. The defect in carotenoid biosynthesis in the Δslr1599 strain under LAHG is reflected by the loss of carotenoid absorbance at ~475 nm and the concomitant appearance of absorbance features in the 350–430 nm region of the absorbance spectrum due to the accumulation of 9,15,9′-tri-*cis*-ζ-carotene (Figure 4B).

We ruled out a polar effect of the disruption of slr1599 by the complementation of the Δslr1599 mutant; the *in trans* expression of slr1599 or the *Arabidopsis Z-ISO* gene (At1g10830 lacking its predicted 58 aa N-terminal chloroplast transit peptide) under the control of the native *psbAII* promoter restored β-carotene biosynthesis under LAHG (Figure S1).

### 3.4. Complementation of the Δslr1599 Mutant Allows Identification of Key Z-ISO Residues

Residues H150, C263, H266, and D294 have previously been implicated as being functionally important in the maize Z-ISO enzyme [5,6]. These residues appear to be highly conserved in Z-ISO homologs (see Figure 2) and we generated point mutants in the equivalent positions in the *Synechocystis* enzyme (H29, C142, H145 and D173) to see if they could complement the Δslr1599 mutant phenotype, as was the case with the *in trans* expression of the WT slr1599 gene discussed above.

**Figure 5:**
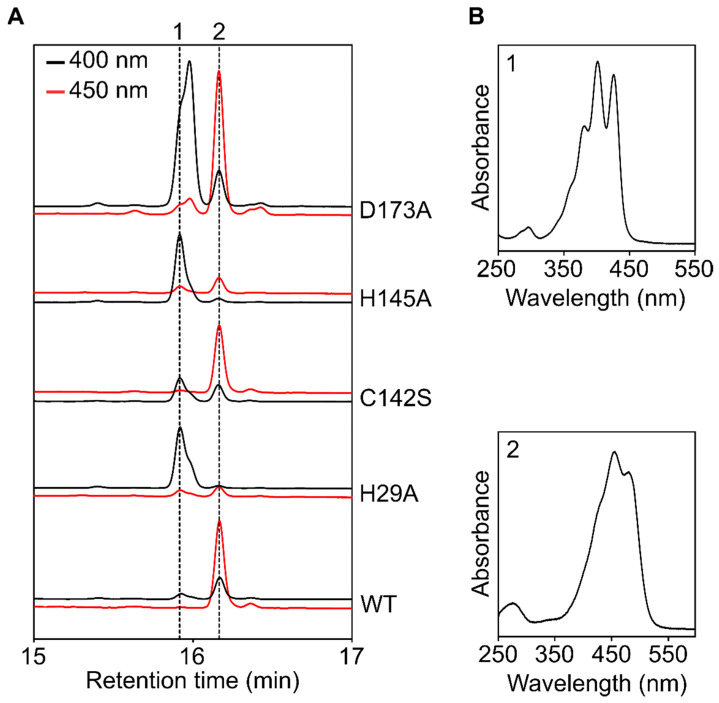
Complementation of the Δslr1599 mutant enables the identification of functionally important residues. (**A**) Analysis of the activity of the indicated Slr1599 variants produced in the Δslr1599 background. Strains were grown under LAHG conditions and carotenoids were extracted and separated by RP-HPLC, and detected by absorbance at 400- and 450 nm. Dotted lines and numbers correspond to the spectra in panel (**B**). (**B**) Absorbance spectra of the carotenoid peaks in panel (**A**). Peak 1 = 9,15,9′-tri-*cis*-ζ-carotene and peak 2 = β-carotene.

As with previous observations of the maize enzyme [5], the *Synechocystis* H29A and H145A substitutions appear to have decreased Z-ISO activity, observed as a large reduction in β-carotene accumulation and the presence of 9,15,9′-tri-*cis*-ζ-carotene under LAHG conditions (Figure 5). Conversely, the C142S variant produced β-carotene but also accumulated some 9,15,9′-tri-*cis*-ζ-carotene (Figure 5), again consistent with the equivalent C263A variant of the maize enzyme [5,6]. Beltrán Zambano previously reported that D294 was essential for the activity of maize Z-ISO [6]; the equivalent D173A mutant of the *Synechocystis* enzyme produced β-carotene but also accumulated a significant amount of a *cis*-ζ-carotene species, suggesting some perturbation of function.

These data demonstrate the utility of the Δslr1599 mutant as a background strain for studying variants of Z-ISO, such as point mutants of the native or heterologous enzymes, or for testing the activity of candidate Z-ISOs from other organisms, in vivo. The functional implications of the results obtained with the H29A, C142S, H145A, and D173A variants of Slr1599 are discussed in relation to the modelled structures of Z-ISO in Section 3.6 below.

### 3.5. Attempted Purification of Recombinant and Native Synechocystis Z-ISO

The maize Z-ISO has previously been purified as a recombinant His-tagged MBP-fusion from detergent-solubilized *E. coli* membranes and shown to contain a heme B cofactor [5,35]. We generated a construct similar to that described by Beltrán Zambano [6] to produce N-terminally His-MBP-tagged maize Z-ISO, and a second construct in which the maize *Z-ISO* gene was replaced with slr1599 (fusion gene sequences are provided in Table S3). Following the protocol described by Wurtzel and Beltrán [35] (see Section 2.4), we were able to isolate the maize protein and demonstrate similar spectroscopic characteristics to those reported previously (Figure S2) [5]. However, we were unable to purify Slr1599 using the same approach; unlike with the maize enzyme, the *E. coli* pellets and membranes from cells carrying the slr1599 construct were not pink/red, indicating that significant quantities of a heme-containing protein were not produced, and the protein was not detected by SDS-PAGE or immunoblot analysis. Attempts to produce the *Synechocystis* protein using a second construct with the gene cloned into the commercial pMAL-c5X plasmid (Table S1) were also unsuccessful.

The maize Z-ISO and other plant enzymes are predicted to have seven TMHs with an N-terminus in/C-terminus out orientation [5,12], whereas the *Synechocystis* enzyme is predicted to contain one less TMH with both the N- and C-termini in the periplasm/thylakoid lumen (Figure 2). While cytoplasmic MBP is compatible with the N-terminal orientation of the maize enzyme, we hypothesized that the presence of MBP may prevent the localization of the N-terminus of the *Synechocystis* enzyme to the periplasm, preventing the proper folding, orientation, and insertion of Z-ISO into the membrane. To retain MBP and the N-terminus of Z-ISO in the cytoplasm and periplasm, respectively, we fused a single TMH protein, YnhF [45], between MBP and Z-ISO. Despite repeated attempts to produce and purify the fusion protein, we were unable to do so. We also inserted slr1599 into the commercial pMAL-p5x vector to N-terminally fuse *Synechocystis* Z-ISO to an MBP variant that is directed to the periplasm so that the N-terminus of Z-ISO is in the correct ‘out’ orientation (Table S1), but this also failed to yield detectable quantities of protein.

Due to the inability to isolate recombinant Slr1599 from *E. coli*, we changed tack and tried to purify the enzyme from the native cyanobacterial host. To attempt to immunoprecipitate Slr1599 from *Synechocystis*, we expressed the gene encoding the enzyme with a 3xFLAG tag from the *psbAII* locus in the Δslr1599 background, generating the strain Δslr1599 FLAG-Slr1599. It was only possible to perform this with the FLAG tag at the N-terminus as we were unable to isolate *Synechocystis* colonies for the C-terminally-tagged equivalent, which is consistent with previous findings that the C-terminus is important for the function of plant Z-ISO [6]. FLAG-tagged Slr1599 was functional as the Δslr1599 FLAG-Slr1599 strain produced β-carotene during LAHG (Figure S1); however, we were unable to purify the tagged enzyme from detergent-solubilized membranes from the cells grown under these conditions or under constant illumination. We speculate that the enzyme is quickly degraded in the light, either during growth under illumination, where it is not required and may be inactivated by the proposed redox-dependent ligand-switching mechanism [5], or during the subsequent cell breakage and purification process, even though these steps were performed under dim light. Consistent with this hypothesis, Slr1599 was not identified in our recent quantitative proteomics analysis of *Synechocystis* cells grown under constant illumination, conditions where PDS, ZDS, and CRT-ISO were all detected [46]. Our inability to isolate Slr1599 following LAHG suggests that the enzyme is present in low amounts even under conditions where we have shown that it is required for carotenoid biosynthesis (Figure 4).

### 3.6. Modelling of Z-ISO for In Silico Structure–Function Analysis

As a structure of Z-ISO remains elusive, we used a combination of AlphaFold [37], Autodock [40], and Rosetta [47,48] to probe the folding homology and structure–function relationships in Slr1599 and the two best-studied plant Z-ISO homologs in silico. While these approaches provide useful information, we stress that the AlphaFold structures, free-energy estimates, and cofactor docking are all computational models and should not be considered replacements for experimentally determined structures, and we note the recent publication of a genomics-based approach to identify Z-ISO orthologs suitable for structural studies [49].

**Figure 6:**
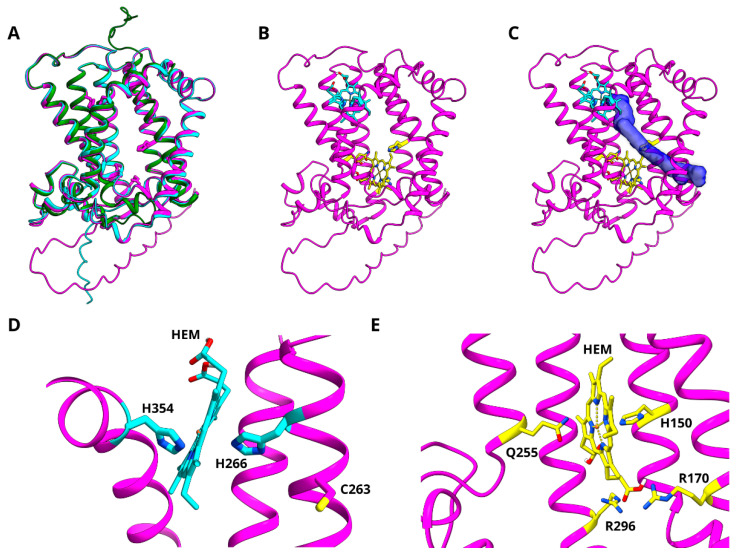
AlphaFold models of Z-ISO. (**A**) Backbone structures of the *Synechocystis* (green), *Arabidopsis* (cyan), and maize (magenta) Z-ISO enzymes. (**B**) Positions of the two putative heme (HEM) binding sites in the maize enzyme, with H266 (cyan) and H150 (yellow) acting as heme ligands. (**C**) Density for the lowest-energy mode identified for 9,15,9′-tri-*cis*-ζ-carotene (purple) binding to maize Z-ISO. (**D**,**E**) Expanded views of the putative H266 (panel (**D**)) and H150 (panel (**E**)) binding sites.

Model structures derived from AlphaFold simulations are available for the maize and *Arabidopsis* Z-ISO enzymes from UniProt (identifiers B4FHU1 and Q9SAC0, respectively) and the Slr1599 model was generated using AlphaFold v2.1.2 (see Section 2.6 for details); all the models have high pLDDT confidence scores and the structural coordinate files and per-residue model confidence values for Slr1599 are available for download in the Supplementary Information. The superimposition of the model structures with the transit signal peptides removed for the plant enzymes shows a very high homology (RMSD 0.4–0.9 Å; Figure 6A). In agreement with the previous predictions, the plant enzymes have seven TMHs and Slr1599 has six, with N- and C-termini on opposing sides of the membrane for the plant Z-ISOs and the same (lumen) side of the membrane for the *Synechocystis* enzyme.

Two histidine residues, H150 and H266, and a cysteine, C263, have previously been implicated in heme binding/Z-ISO activity in work with the maize enzyme and these residues are conserved in Z-ISO homologs (Figure 2 and Figure S3). Mutagenesis studies showed that H150 and H266 are the only histidine residues required for function in maize Z-ISO [5] and we found the equivalent H29 and H145 residues are important for activity in Slr1599 (Figure 5). Based on homology modelling with maize Z-ISO, Beltràn et al proposed that H150 acts as the proximal ligand to the heme cofactor and H266 and C263 act as alternate distal heme ligands in a redox-regulated ligand-switching mechanism that activates/inactivates the enzyme [5]. However, the AlphaFold models predict that H150 and H266 are too far apart in the structures for bis-His coordination of the heme. We therefore used Autodock Vina to dock heme B into the maize and *Arabidopsis* Z-ISO models to investigate possible alternatives for heme ligation.

First, a very broad search area (125,000 Å^3^) was used for both structures (see Section 2.6 for details). In *Arabidopsis*, four potential heme binding sites were predicted, two of which were of particular interest (Figure S4); one site is oriented near H150 and another in close proximity to H266 (maize enzyme numbering). The H266 site contains a second possible axial ligand to coordinate the heme Fe-center, H354, and is proximal to the suggested alternative heme-coordinating residue, C263 [5], however we note that H354 was not required for Z-ISO function in previous studies [5,6]. Conversely, in the other site there is no residue to donate the requisite sixth coordinate bond to the Fe center, suggesting that H150 is involved in inter-domain H-bonding and has a structural role rather than facilitating cofactor binding (Figure S8B).

In the broad search regime, with the same sampling method used, Autodock Vina did not find the equivalent H266/H354 bis-His binding site in the maize enzyme model. This is possibly due to the rotamer position of H354 in the maize structure being more directly aligned with H266 (Figure S5), leading to a narrower binding cavity that is difficult to identify in the simulation. Given that heme binding would likely occur through axial ligation, a narrower sampling area (2744 Å^3^) was used for the maize enzyme focusing on the H266 site. Under these conditions, the heme B group was reproducibly positioned with the central Fe positioned approximately between H266 and H354, albeit with a higher binding energy for all modes than the alternative H150 site (Figure S6). The lowest energy binding modes from each of the two putative heme binding sites are displayed for the maize structural model in Figure 6B,D,E.

The model with the highest-affinity binding mode for the putative H266/H354 site was subsequently used to dock 9,15,9′-tri-*cis*-ζ-carotene to investigate whether the putative cofactor binding site was close to the carotenoid substrate, thereby identifying a possible active site. The twenty binding modes identified in the simulation fell broadly into three groups of similar orientation, all with similar binding energies (Figure S7). One of these groups positions the substrate in proximity to both the predicted position of the heme B cofactor and the H150 residue and is displayed in Figure 6C. The proximity of H150 to the 15-*cis* bond of 9,15,9′-tri-*cis*-ζ-carotene and its known involvement in Z-ISO activity suggests that this residue may have a catalytic role in the *cis*–to-*trans* isomerization. A conserved serine (S177 in maize) is located ~3 Å away from H150, suggesting these two residues may form a catalytic dyad (Figure S8B).

Finally, D294 has also been implicated in Z-ISO activity [6]. The AlphaFold structures identified possible H-bond networks from this residue to residues on other secondary motifs, suggesting a role for this aspartate in protein folding (Figure S7A). Alanine substitutions of D294 and its predicted H-bond partners R252 and H253 gave increased free energy predictions relative to the WT protein (Figure S8A).

### 3.7. In Silico Mutagenesis and Biochemical Characterization of Putative Heme Binding Sites

We used the Rosetta score function to estimate free energy changes following the in silico mutagenesis of the two putative heme-binding sites (H150 and H266) in the maize Z-ISO AlphaFold model. Unlike AlphaFold, which scores protein-folding accuracy based on an internal confidence probability derived from the machine learning procedure, Rosetta uses a scoring system related to thermodynamic free-energy changes (known as Rosetta energy units, REU) based on calculations of physiologically relevant parameters [39]. The H150A substitution resulted in a small increase in relative free energy compared to WT (−1104 and −1109 REU, respectively), possibly indicating that this substitution alters protein folding, which may explain the decreased and altered heme binding by this mutant observed previously [5]. No inter-domain interactions were identified at the H266 site and the H266A substitution resulted in only a marginal free energy decrease relative to WT (−1110 and 1109 REU, respectively), likely due to the preference for hydrophobic residue packing in the absence of a cofactor. 

**Figure 7:**
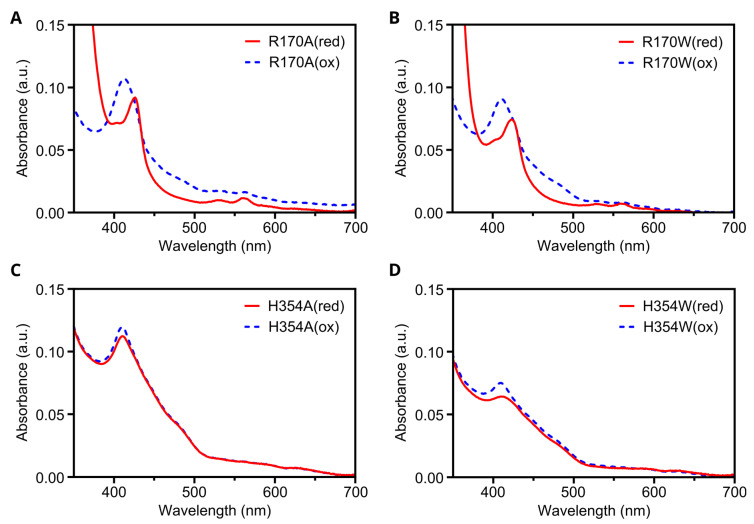
Oxidized and reduced spectra of maize Z-ISO mutants. MBP-*Zm*Z-ISO Arg170Ala (**A**), Arg170Trp (**B**), His354Ala (**C**), and His354Trp (**D**) proteins produced in *E. coli* were solubilized in β-DDM and partially purified by amylose affinity chromatography. The oxidized (ox—dotted blue trace) and reduced (red—solid red trace) absorbance spectra of the eluates were measured after addition of potassium ferricyanide or sodium dithionite, respectively.

We further investigated the prospective heme binding sites by performing in silico mutagenesis around the H150 and H266 residues and estimating the free-energy changes using Rosetta. We sought to change the chemical nature of residues (e.g., His to Ala) or introduce a steric clash (e.g., His to Trp) to block the binding cavity. We also aimed to maintain the protein structure using mutations that would have a minimal effect on folding (i.e., minor free-energy changes). For the H266 site, H354 (approximately axial to H266; Figure 6D) was mutated to Ala or Trp, with relatively minor free-energy changes (+1 and +5 REU; Figure S8C). For the H150 site, we analyzed mutations to three proximal residues (Figure 6E), R170, Q255, and R296, changing each to Ala, Phe, or Trp. The Ala and Trp substitution in R170 yielded the most minimal free-energy changes of the three residues (Figure S8C). Thus, the H354A, H354W, R170A, and R170W variants were selected for production in *E. coli*. The membrane extracts and crude protein preparations were visible colored for all four of these protein variants, suggesting that they all bound at least some heme. A comparison of the oxidized and reduced spectrum of the R170A and R170W crude protein extracts showed a typical shift upon the addition of sodium dithionite, suggesting that neither of these substitutions prevent heme binding to Z-ISO (Figure 7A,B). Although pigmentation was observed in the H354A and H354W mutants, no significant change was observed between the oxidized and reduced spectra (Figure 7C,D) [5]. This suggests that heme may be weakly bound by the remaining histidine ligand (H266), but without the requisite sixth coordination bond, the addition of sodium dithionite is insufficient to reduce the ferric center, and hence no spectral changes are observed. In combination with the above modeling, these preliminary results suggest that H266 and H354 are likely to coordinate heme B, while H150 may instead have a structural or catalytic role.

## 4. Conclusions

In this study, we have shown that Slr1599 is the *Synechocystis* Z-ISO, and that it is required for the *cis*-to-*trans* isomerization of the central 15–15′ *cis* double bond of 9,15,9′-tri-*cis*-ζ-carotene for the synthesis of carotenoids when the organism is grown in the absence of light. The experimental verification of Z-ISO completes the biosynthetic pathway from phytoene to lycopene, the common precursor of β-carotene and all the xanthophylls utilized for light absorption, energy transfer, and photoprotection, in this model cyanobacterium. In addition to revealing the phenotype of a cyanobacterial Δ*Z-ISO* mutant, we have demonstrated the utility of the Δslr1599 strain for in vivo testing of key residues or the confirmation of Z-ISO homologs from other organisms.

Despite adopting numerous approaches, we were unable to purify Slr1599 for spectroscopic or structural analysis, either as a recombinant MBP-fusion protein from *E. coli* or with a FLAG-tag from the native host. However, sequence alignments and site-directed mutagenesis of conserved residues suggest that Slr1599 contains a heme cofactor like the well-characterized maize enzyme [5]. We suggest that the heme has a role in redox sensing, where the ligand-switch activation model proposed by Beltrán and colleagues [5] would allow for Z-ISO to be inactivated when *Synechocystis* is grown under illumination. Under these conditions, Z-ISO activity can be replaced by photo-isomerization, and we predict that Z-ISO is quickly degraded as we were not able to detect the protein in the proteome of photoautotrophically grown cells [46]. Our failure to isolate the protein during LAHG further suggests that the protein is present at very low levels, even under conditions where we have shown that its activity is required for carotenogenesis.

In the absence of a structure of Z-ISO, we used AlphaFold models and in silico docking to investigate the heme- and substrate-binding sites of Slr1599 and the Z-ISO homologs from maize and *Arabidopsis*. The models suggest that the H150 (maize numbering) may not act as a heme ligand as previously suggested [5], but its position relative to the predicted substrate-binding site offers an explanation for why its substitution with alanine results in a loss of activity in both the maize and *Synechocystis* enzymes. The minimally disruptive mutagenesis strategy and preliminary biochemical analysis described here suggests that it is instead H354 that acts alongside H266 to ligate the heme B cofactor. However, we emphasize that our in silico analysis is not a substitute for an experimentally determined structure of Z-ISO and greater understanding of this complex enzyme requires further experimental work.

## Data Availability

Where not included in the article or supplementary material, the data presented in this study are available on request from the corresponding author.

## Supplementary Materials

The following supporting information can be downloaded at: https://www.mdpi.com/article/10.3390/microorganisms10091730/s1. Table S1: Plasmids used in this study; Table S2: Primers used in this study; Table S3: Sequences of MBP-Z-ISO fusion genes used in this study; Table S4: ROSETTA membrane protein scores for abolishment of putative H150 binding site in *Zea mays* Z-ISO; Figure S1: Complementation of the *Synechocystis* Δslr1599 mutant; Figure S2: Purification of the *Zea mays* (*Zm*) MBP-Z-ISO fusion protein; Figure S3: Orientation of conserved Z-ISO residues with putative catalytic activity; Figure S4: Heme B docking in *Arabidopsis* (A) and maize (B) Z-ISO using Autodock Vina; Figure S5: Alignment of H266 and H354, relative to C263, in maize (magenta) and *Arabidopsis* (cyan) Z-ISO; Figure S6: Heme docking at the H266/H354 site in maize Z-ISO; Figure S7: Docking 9,15,9′-tri-*cis*-ζ-carotene into the maize Z-ISO-heme B model; Figure S8: Using the Rosetta score function to estimate free-energy changes of point mutations. File S1 (Slr1599.pdb): AlphaFold structure file for Slr1599.

## References

[1] Cogdell R.J., Frank H.A. (1987). How Carotenoids Function in Photosynthetic Bacteria. Biochim. Biophys. Acta.

[2] Hashimoto H., Uragami C., Cogdell R.J. (2016). Carotenoids and Photosynthesis. Subcell. Biochem..

[3] Rosas-Saavedra C., Stange C. (2016). Biosynthesis of Carotenoids in Plants: Enzymes and Color. Subcell. Biochem..

[4] Canniffe D.P., Hitchcock A., Jez J. (2021). Carotenoids in photosynthesis—Structure and biosynthesis. Encyclopedia of Biological Chemistry III.

[5] Beltrán J., Kloss B., Hosler J.P., Geng J., Liu A., Modi A., Dawson J.H., Sono M., Shumskaya M., Ampomah-Dwamena C. (2015). Control of carotenoid biosynthesis through a heme-based *cis*-*trans* isomerase. Nat. Chem. Biol..

[6] Beltràn Zambrano J.A. (2015). Functional Characterization of The Plant 15-Cis-Zeta-Carotene Isomerase Z-Iso. https://academicworks.cuny.edu/gc_etds/521.

[7] Li F., Murillo C., Wurtzel E.T. (2007). Maize *Y9* encodes a product essential for 15-*cis*-zeta-carotene isomerization. Plant Physiol..

[8] Chen Y., Li F., Wurtzel E.T. (2010). Isolation and characterization of the *Z-ISO* gene encoding a missing component of carotenoid biosynthesis in plants. Plant Physiol..

[9] Liu X., Hu Q., Yan J., Sun K., Liang Y., Jia M., Meng X., Fang S., Wang Y., Jing Y. (2020). ζ-Carotene Isomerase Suppresses Tillering in Rice through the Coordinated Biosynthesis of Strigolactone and Abscisic Acid. Mol. Plant.

[10] Zhou H., Yang M., Zhao L., Zhu Z., Liu F., Sun H., Sun C., Tan L. (2021). HIGH-TILLERING AND DWARF 12 modulates photosynthesis and plant architecture by affecting carotenoid biosynthesis in rice. J. Exp. Bot..

[11] Sugiyama K., Takahashi K., Nakazawa K., Yamada M., Kato S., Shinomura T., Nagashima Y., Suzuki H., Ara T., Harada J. (2020). Oxygenic Phototrophs Need ζ-Carotene Isomerase (Z-ISO) for Carotene Synthesis: Functional Analysis in *Arthrospira* and *Euglena*. Plant Cell Physiol..

[12] Rodrigo M.J., Lado J., Alós E., Alquézar B., Dery O., Hirschberg J., Zacarías L. (2019). A mutant allele of ζ-carotene isomerase (*Z-ISO*) is associated with the yellow pigmentation of the “Pinalate” sweet orange mutant and reveals new insights into its role in fruit carotenogenesis. BMC Plant Biol..

[13] Efremov G.I., Shchennikova A.V., Kochieva E.Z. (2021). Characterization of 15-*cis*-ζ-Carotene Isomerase Z-ISO in Cultivated and Wild Tomato Species Differing in Ripe Fruit Pigmentation. Plants.

[14] Fantini E., Falcone G., Frusciante S., Giliberto L., Giuliano G. (2013). Dissection of tomato lycopene biosynthesis through virus-induced gene silencing. Plant Physiol..

[15] Gisriel C.J., Wang J., Liu J., Flesher D.A., Reiss K.M., Huang H.L., Yang K.R., Armstrong W.H., Gunner M.R., Batista V.S. (2022). High-resolution cryo-electron microscopy structure of photosystem II from the mesophilic cyanobacterium, *Synechocystis* sp. PCC 6803. Proc. Natl. Acad. Sci. USA.

[16] Malavath T., Caspy I., Netzer-El S.Y., Klaiman D., Nelson N. (2018). Structure and function of wild-type and subunit-depleted photosystem I in *Synechocystis*. Biochim. Biophys. Acta. Bioenerg..

[17] Kurisu G., Zhang H., Smith J.L., Cramer W.A. (2003). Structure of the cytochrome *b*_6_*f* complex of oxygenic photosynthesis: Tuning the cavity. Science.

[18] Proctor M.S., Malone L.A., Farmer D.A., Swainsbury D.J.K., Hawkings F.R., Pastorelli F., Emrich-Mills T.Z., Siebert C.A., Hunter C.N., Johnson M.P. (2022). Cryo-EM structures of the *Synechocystis* sp. PCC 6803 cytochrome *b*_6_*f* complex with and without the regulatory PetP subunit. Biochem. J..

[19] Schuller J.M., Birrell J.A., Tanaka H., Konuma T., Wulfhorst H., Cox N., Schuller S.K., Thiemann J., Lubitz W., Sétif P. (2019). Structural adaptations of photosynthetic complex I enable ferredoxin-dependent electron transfer. Science.

[20] Schuller J.M., Saura P., Thiemann J., Schuller S.K., Gamiz-Hernandez A.P., Kurisu G., Nowaczyk M.M., Kaila V.R. (2020). Redox-coupled proton pumping drives carbon concentration in the photosynthetic complex I. Nat. Commun..

[21] Bautista J.A., Rappaport F., Guergova-Kuras M., Cohen R.O., Golbeck J.H., Wang J.Y., Béal D., Diner B.A. (2005). Biochemical and biophysical characterization of photosystem I from phytoene desaturase and zeta-carotene desaturase deletion mutants of *Synechocystis* Sp. PCC 6803: Evidence for PsaA- and PsaB-side electron transport in cyanobacteria. J. Biol. Chem..

[22] Breitenbach J., Fernández-González B., Vioque A., Sandmann G. (1998). A higher-plant type zeta-carotene desaturase in the cyanobacterium *Synechocystis* PCC6803. Plant Mol. Biol..

[23] Masamoto K., Wada H., Kaneko T., Takaichi S. (2001). Identification of a gene required for *cis*-to-*trans* carotene isomerization in carotenogenesis of the cyanobacterium *Synechocystis* sp. PCC 6803. Plant Cell Physiol..

[24] Tóth T.N., Chukhutsina V., Domonkos I., Knoppová J., Komenda J., Kis M., Lénárt Z., Garab G., Kovács L., Gombos Z. (2015). Carotenoids are essential for the assembly of cyanobacterial photosynthetic complexes. Biochim. Biophys. Acta.

[25] Masamoto K., Hisatomi S., Sakurai I., Gombos Z., Wada H. (2004). Requirement of carotene isomerization for the assembly of photosystem II in *Synechocystis* sp. PCC 6803. Plant Cell Physiol..

[26] Isaacson T., Ohad I., Beyer P., Hirschberg J. (2004). Analysis *in vitro* of the enzyme CRTISO establishes a poly-cis-carotenoid biosynthesis pathway in plants. Plant Physiol..

[27] Cunningham F.X., Gantt E. (2007). A portfolio of plasmids for identification and analysis of carotenoid pathway enzymes: *Adonis aestivalis* as a case study. Photosynth. Res..

[28] Proctor M.S., Pazderník M., Jackson P.J., Pilný J., Martin E.C., Dickman M.J., Canniffe D.P., Johnson M.P., Hunter C.N., Sobotka R. (2020). Xanthophyll carotenoids stabilise the association of cyanobacterial chlorophyll synthase with the LHC-like protein HliD. Biochem. J..

[29] Rippka R., Derueles J., Waterbury J.B., Herdman M., Stainer R.Y. (1979). Generic Assignments, Strain Histories and Properties of Pure Cultures of Cyanobacteria. J. Gen. Microbiol..

[30] Anderson S.L., McIntosh L. (1991). Light-activated heterotrophic growth of the cyanobacterium *Synechocystis* sp. strain PCC 6803: A blue-light-requiring process. J. Bacteriol..

[31] Hitchcock A., Jackson P.J., Chidgey J.W., Dickman M.J., Hunter C.N., Canniffe D.P. (2016). Biosynthesis of Chlorophyll *a* in a Purple Bacterial Phototroph and Assembly into a Plant Chlorophyll–Protein Complex. ACS Synth. Biol..

[32] Hollingshead S., Kopečná J., Jackson P.J., Canniffe D.P., Davison P.A., Dickman M.J., Sobotka R., Hunter C.N. (2012). Conserved chloroplast open-reading frame *ycf54* is required for activity of the magnesium protoporphyrin monomethylester oxidative cyclase in *Synechocystis* PCC 6803. J. Biol. Chem..

[33] Chen G.E., Hitchcock A., Mareš J., Gong Y., Tichý M., Pilný J., Kovářová L., Zdvihalová B., Xu J., Hunter C.N. (2021). Evolution of Ycf54-independent chlorophyll biosynthesis in cyanobacteria. Proc. Natl. Acad. Sci. USA.

[34] Chidgey J.W., Linhartová M., Komenda J., Jackson P.J., Dickman M.J., Canniffe D.P., Koník P., Pilný J., Hunter C.N., Sobotka R. (2014). A cyanobacterial chlorophyll synthase-HliD complex associates with the Ycf39 protein and the YidC/Alb3 insertase. Plant Cell.

[35] Wurtzel E.T., Beltrán J. (2020). Improved Expression and Purification of the Carotenoid Biosynthetic Enzyme Z-ISO. Methods Mol. Biol..

[36] Beltrán J., Wurtzel E.T. (2022). Enzymatic isomerization of ζ-carotene mediated by the heme-containing isomerase Z-ISO. Methods Enzymol..

[37] Jumper J., Evans R., Pritzel A., Green T., Figurnov M., Ronneberger O., Tunyasuvunakool K., Bates R., Žídek A., Potapenko A. (2021). Highly accurate protein structure prediction with AlphaFold. Nature.

[38] Pettersen E.F., Goddard T.D., Huang C.C., Meng E.C., Couch G.S., Croll T.I., Morris J.H., Ferrin T.E. (2021). UCSF ChimeraX: Structure visualization for researchers, educators, and developers. Protein Sci..

[39] Alford R.F., Leaver-Fay A., Jeliazkov J.R., O’Meara M.J., DiMaio F.P., Park H., Shapovalov M.V., Renfrew P.D., Mulligan V.K., Kappel K. (2017). The Rosetta All-Atom Energy Function for Macromolecular Modeling and Design. J. Chem. Theory Comput..

[40] Trott O., Olson A.J. (2010). AutoDock Vina: Improving the speed and accuracy of docking with a new scoring function, efficient optimization, and multithreading. J. Comput. Chem..

[41] Zhu Y., Graham J.E., Ludwig M., Xiong W., Alvey R.M., Shen G., Bryant D.A. (2010). Roles of xanthophyll carotenoids in protection against photoinhibition and oxidative stress in the cyanobacterium *Synechococcus* sp. strain PCC 7002. Arch. Biochem. Biophys..

[42] Kaneko T., Sato S., Kotani H., Tanaka A., Asamizu E., Nakamura Y., Miyajima N., Hirosawa M., Sugiura M., Sasamoto S. (1996). Sequence analysis of the genome of the unicellular cyanobacterium *Synechocystis* sp. strain PCC 6803. II. Sequence determination of the entire genome and assignment of potential protein-coding regions. DNA Res..

[43] Trautner C. (2011). Synechocystis mutants lacking genes potentially involved in carotenoid metabolism. https://keep.lib.asu.edu/items/149541.

[44] Niedzwiedzki D.M., Swainsbury D.J.K., Canniffe D.P., Hunter C.N., Hitchcock A. (2020). A photosynthetic antenna complexes foregoes unity carotenoid to bacteriochlorophyll energy transfer efficiency to ensure photoprotection. Proc. Natl. Acad. Sci. USA.

[45] Fontaine F., Fuchs R.T., Storz G. (2011). Membrane localization of small proteins in *Escherichia coli*. J. Biol. Chem..

[46] Jackson P.J., Hitchcock A., Brindley A.A., Dickman M.J., Hunter C.N. (2022). Absolute quantification of cellular levels of photosynthesis-related proteins in *Synechocystis* sp. PCC 6803. Photosynth. Res..

[47] Rohl C.A., Strauss C.E.M., Misura K.M.S., Baker D. (2004). Protein Structure Prediction Using Rosetta. Methods Enzymol..

[48] Das R., Baker D. (2008). Macromolecular modeling with rosetta. Annu. Rev. Biochem..

[49] Kloss B. (2022). Genomics-based strategies toward the identification of a Z-ISO carotenoid biosynthetic enzyme suitable for structural studies. Methods Enzymol..

