# Supplementary material for: Zeta-Carotene Isomerase (Z-ISO) Is Required for Light-Independent Carotenoid Biosynthesis in the Cyanobacterium Synechocystis sp. PCC 6803
Source: Microorganisms. 2022 Aug 27;10(9):1730. doi: 10.3390/microorganisms10091730 (PMC9505123; doi:10.3390/microorganisms10091730)
Supplement: Supplementary file 1 [file microorganisms-10-01730-s001.zip › microorganisms-1867373-supplementary.pdf]

Supplementary Information for: Proctor *et al*. Zeta-carotene isomerase (Z-ISO) is required for light-independent carotenoid biosynthesis in the cyanobacterium *Synechocystis* sp. PCC 6803

Table S1. Plasmids used in this study.

Plasmid name	Details	Source/reference[1]
pCDFDuet^{Tm}-1	Has two multiple cloning sites (MCS) for IPTG-inducible coexpression of two genes. AmpR.	Novagen
pAC-ZETAipi	Contains *crtE*, *crtB*, and *idi* genes of *Erwinia herbicola* (*Pantoea agglomerans*) Eho10, and *pds* (*crtP*) gene of *Synechococcus* PCC 7942 to produce zeta-carotene in *E. coli.* CmR.	Addgene; Cunningham and Gantt, 2007 [27]
pPD-*N*FLAG	Genes cloned into NotI/BglII sites are preceded by sequence encoding N-terminal 3xFLAG tag. Integrates into *Synechocystis* genome at *psbAII* locus. KanR.	Hollingshead et al. 2012 [32]
pPD-*C*FLAG	Genes cloned into NdeI/NheI sites are followed by sequence encoding C-terminal 3xFLAG tag. Integrates into *Synechocystis* genome at *psbAII* locus. KanR.	Chidgey et al. 2014 [34]
pET21a(+)	Plasmid for IPTG inducible gene expression. AmpR.	Novagen
pMAL-c5x	Allows inducible cytoplasmic expression of maltose-binding protein (MBP) fusions with a Factor Xa cleavage site. AmpR.	New England Biolabs
pMAL-p5x	Allows inducible periplasmic expression of maltose-binding protein (MBP) fusions with a Factor Xa cleavage site. AmpR.	New England Biolabs
pAH592	slr1599 inserted at MCS2 of pCDFDuet^{Tm}-1.	This study
pAH595	Maize *Z-ISO* inserted at MCS2 of pCDFDuet^{Tm}-1.	This study
pAH585	*Synechocystis zds* (*crtQ*; *slr0940*) inserted at MCS1 of pCDFDuet^{Tm}-1.	This study
pAH587	slr1599 inserted at MCS2 of pAH585.	This study
pAH590	Maize *Z-ISO* inserted at MCS2 of pAH585.	This study
pAH445	Slr1599 inserted into NotI/BglII sites of pPD-*N*FLAG	This study
pAH436	*Arabidopsis Z-ISO* gene (At1g10830) lacking the first 174 bp (the sequence encoding the N-terminal chloroplast transit peptide) and with the sequence encoding a Ser-Ala linker	This study

	followed by the Strep-tag® II added to the 3′ end of the gene inserted into NdeI/BglII sites of pPD-*N*FLAG	
pAH545	Codon optimized His-MBP-*ZmZ-ISO* construct inserted into pET21a(+)	This study
pAH546	As pAH545 but with the *ZmZ-ISO* replaced by slr599.	This study
pAH565	As pAH546 but with *ynhF* inserted upstream of slr1599.	This study
pAH424	slr1599 inserted into pMAL-c5x	This study
pAH707	slr1599 inserted into pMAL-p5x	This study

[1]See main paper for references.

Table S2. Primers used in this study. Restriction sites are shown bold italics, mismatches in QuikChange primers are underlined and homology regions for OLE-PCR are underlined and bold.

Primer	Sequence (5´→ 3´)	Details
AH1203<br>AH1206	AAGCCT***CATATG***TTGTTTGATATTTCTTCTCCTTGGC<br>AGCAAT***CTCGAG***CTACCAATTAACTCTGGAGGTC	Primer pair for slr1599 into pCDFDuet MCS2 (NdeI/XhoI)
AH1207<br>AH1208	AAGCCT***CCATGG***GGCGTGTTGCAATTGTGGGAG<br>AGCAAT***GTCGAC***TTACTGAGATGCCAGGACTG	Primer pair for *SynZDS* into pCDFDuet MCS1 (NcoI/SalI)
AH912<br>AH913<br>AH914<br>AH915	TAGAACCAACGATTGACCAATTCAC<br>**CTGCAATCGGATGCGATTATTGAATA**ATGGCAAACAACAGAAGGTAGG<br>**GCATCCCTTAACTTGTTTTTCGTGTG**TGGGGGTAATGGGTTTCATTGC<br>TTGCATTTGTTGCAGGGCTTGG	Primers for generation of slr1599 deletion construct by OLE-PCR (912/913 UsF and 914/915 DsF)
AH595<br>AH596	TATTCAATAATCGCATCCGATTGCAG<br>CACACGAAAAACAAGTTAAGGGATGC	Primer pair for erythromycin resistance cassette
AH955<br>AH956	AAGCCT***GCGGCCGC***ATTTGATATTTCTTCTCCTTGGC<br>AGCAAT***AGATCT***CTACCAATTAACTCTGGAGGTCATC	Primer pair for *Syn*Z-ISO into pPD-*N*FLAG (NotI/BglII)
AH957<br>AH958	AAGCCT***CATATG***TTTGATATTTCTTCTCCTTGGC<br>AGCAAT***GCTAGC***CCAATTAACTCTGGAGGTCATCAC	Primer pair for *Syn*Z-ISO into pPD-*C*FLAG (NdeI/NheI)
AH198<br>AH199	GGTATATGGATCATAATTGTATGCCCG<br>CTATTCAATCGCTCTAGGCGATCG	Primer pair to amplify *psbAII* region from pPD-*N*/*C*FLAG
AH47<br>AH48	AAACGCCCTCTGTTTACCCA<br>TCAACCCGGTACAGAGCTTC	Primer pair for screening segregation of the *psbAII* locus
AH959<br>AH960	TTCTGTTGTTTGCCATCGTTGCTAGTGGCTTAGCCGCCCTGC<br>AGGGCGGCTAAGCCACTAGCAACGATGGCAAACAACAGAAGG	Primer pair for slr1599 H29A mutation
AH961<br>AH962	TCGGTCAAGTAATTTGGTCCATTGCCCACACCCTCTGG<br>ACCAGAGGGTGTGGGCAATGGACCAAATTACTTGACCGACC	Primer pair for slr1599 C142S mutation
AH963<br>AH964	AGTAATTTGGTGCATTGCCGCCACCCTCTGGTTGGGAACC<br>TGGTTCCCAACCAGAGGGTGGCGGCAATGCACCAAATTACTTGACC	Primer pair for slr1599 H145A mutation
AH965<br>AH966	TTTGCAGTCTGGCATGGCGCTCGCCGTTGGCAACACAAATATG<br>TTGTGTTGCCAACGGCGAGCGCCATGCCAGACTGCAAAGC	Primer pair for slr1599 D173A mutation
MP487<br>MP488	TGACTTTGGGCGCATATTTCGCTGCCCCTCTTATGCAGG<br>CCTGCATAAGAGGGGCAGCGAAATATGCGCCCAAAGTCA	Primer pair for MBP-*Zm*Z-ISO H354A
MP489<br>MP490	CTTTGGGCGCATATTTCGCTTGGCCTCTTATGCAGGCCTC<br>GAGGCCTGCATAAGAGGCCAAGCGAAATATGCGCCCAAAG	Primer pair for MBP-*Zm*Z-ISO H354W

MP491<br>MP492	CGTAGGGGAACGGGTGTATGCCGTGCTGTTC<br>GAACAGCACGGCATACACCCGTTCCCCTACG	Primer pair for MBP-*Zm*Z-ISO R170A
MP493<br>MP494	GGGGAACGGGTGTATTGGGTGCTGTTCGCTG<br>CAGCGAACAGCACCCAATACACCCGTTCCCC	Primer pair for MBP-*Zm*Z-ISO R170W
AH923<br>AH924	AAGCCT***CATATG***TTTGATATTTCTTCTCCTTGGC<br>AGCAAT***GTCGAC***CTACCAATTAACTCTGGAGGTCATC	Primer pair for slr1599 into pMAL-c/p5X (NdeI/SalI)

Table S3. Sequences of MBP-Z-ISO fusion genes used in this study.

Name and sequence	Key
**MBP-Maize Z-ISO fusion:**<br><br>ATGCATCACCACCATCATCATCACCATCATCATtcttctggtgtagatctgggtaccA<br>AAATCGAAGAAGGTAAACTGGTAATCTGGATTAACGGCGATAAAGGCTATAACGGTCT<br>CGCTGAAGTCGGTAAGAAATTCGAGAAAGATACCGGAATTAAAGTCACCGTTGAGCAT<br>CCGGATAAACTGGAAGAGAAATTCCCACAGGTTGCGGCAACTGGCGATGGCCCTGACA<br>TTATCTTCTGGGCACACGACCGCTTTGGTGGCTACGCTCAATCTGGCCTGTTGGCTGA<br>AATCACCCCGGACAAAGCGTTCCAGGACAAGCTGTATCCGTTTACCTGGGATGCCGTA<br>CGTTACAACGGCAAGCTGATTGCTTACCCGATCGCTGTTGAAGCGTTATCGCTGATTT<br>ATAACAAAGATCTGCTGCCGAACCCGCCAAAAACCTGGGAAGAGATCCCGGCGCTGGA<br>TAAAGAACTGAAAGCGAAAGGTAAGAGCGCGCTGATGTTCAACCTGCAAGAACCGTAC<br>TTCACCTGGCCGCTGATTGCTGCTGACGGGGGTTATGCGTTCAAGTATGAAAACGGCA<br>AGTACGACATTAAAGACGTGGGCGTGGATAACGCTGGCGCGAAAGCGGGTCTGACCTT<br>CCTGGTTGACCTGATTAAAAACAAACACATGAATGCAGACACCGATTACTCCATCGCA<br>GAAGCTGCCTTTAATAAAGGCGAAACAGCGATGACCATCAACGGCCCGTGGGCATGGT<br>CCAACATCGACACCAGCAAAGTGAATTATGGTGTAACGGTACTGCCGACCTTCAAGGG<br>TCAACCATCCAAACCGTTCGTTGGCGTGCTGAGCGCAGGTATTAACGCCGCCAGTCCG<br>AACAAAGAGCTGGCAAAAGAGTTCCTCGAAAACTATCTGCTGACTGATGAAGGTCTGG<br>AAGCGGTTAATAAAGACAAACCGCTGGGTGCCGTAGCGCTGAAGTCTTACGAGGAAGA<br>GTTGGCGAAAGATCCACGTATTGCCGCCACCATGGAAAACGCCCAGAAAGGTGAAATC<br>ATGCCGAACATCCCGCAGATGTCCGCTTTCTGGTATGCCGTGCGTACTGCGGTGATCA<br>ACGCCGCCAGCGGTCGTCAGACTGTCGATGAAGCCCTGAAAGACGCGCAGACTgatta<br>cgatatcccaggtaccGAGAACCTGTACTTCCAATCCaatgccatgCGTCCGGCGCGT<br>GCGGTGGGCGGCGGCATTGAACCGAAAGAAGGCGTGGTGGCGGAAGGCGATGAAAGCG<br>GCGGCGGCCCGGTGCTGGTGGGCGAAGATAGCGCGGCGTTTGAACTGAAAGATCAGAG<br>CGTGGCGAGCTGGGCGTATTTTGCGGGCATTCTGGGCGCGGTGCTGGTGGCGCTGAAC<br>GTGCTGTGGATTGATCCGAGCACCGGCGTGGGCACCAAATTTCTGGATGCGGTGGCGA<br>GCGTGAGCGATAGCCATGAAGTGGTGATGCTGCTGCTGACCATTATTTTTGCGGTGGT<br>GCATAGCGGCATGGCGAGCCTGCGTGAAAGCGGCGAAAAAATTGTGGGCGAACGTGTG<br>TATCGTGTGCTGTTTGCGGGCATTAGCCTGCCGCTGGCGGTGACCACCATTGTGTATT<br>TTATTAACCATCGTTATGATGGCACCCAGCTGTGGCAGGTGCAGGGCATTACCGGCAT<br>TCATGAACTGCTGTGGTTTAGCAGCTTTATTAGCTTTTTTTTTCTGTATCCGAGCACC<br>TTTAACCTGCTGGAAGTGGCGGCGGTGGATAAACCGAAACTGCACATGTGGGAAACCG<br>GCATTATGCGTATTACCCGTCATCCGCAGATGGTGGGCCAGGTGATTTGGTGCCTGGC<br>GCATACCCTGTGGATTGGCAATAGCGTGGCGGTGGCGGCGAGCGTGGGCCTGATTAGC<br>CATCATCTGTTTGGCGCGTGGAACGGCGATCGTCGTCTGCTGAGCCGTTATGGCGAAG<br>CGTTTGAAGTGCTGAAAAAACGTACCAGCGTGATGCCGTTTGCGGCGATTATTGATGG<br>CCGTCAGAAACTGCCGAAAGATTATCATAAAGAATTTTTCGTCTGCCGTATGTGGCG<br>ATTACCATGCTGACCCTGGGCGCGTATTTTGCGCATCCGCTGATGCAGGCGAGCAGCT<br>ATCAGCTGCCGTGGTAA	Construct encodes following protein: His$_{10}$-tag-linker-MBP-linker-TEV cleavage site-linker-*Zm*Z-ISO*<br><br>*starting at Arg49 codon and codon optimized for *E. coli* B strain using Integrated DNA Technologies tool
**MBP-*Synechocystis* Z-ISO fusion version 1:**<br><br>ATGCATCACCACCATCATCATCACCATCATCATtcttctggtgtagatctgggtaccA<br>AAATCGAAGAAGGTAAACTGGTAATCTGGATTAACGGCGATAAAGGCTATAACGGTCT<br>CGCTGAAGTCGGTAAGAAATTCGAGAAAGATACCGGAATTAAAGTCACCGTTGAGCAT<br>CCGGATAAACTGGAAGAGAAATTCCCACAGGTTGCGGCAACTGGCGATGGCCCTGACA<br>TTATCTTCTGGGCACACGACCGCTTTGGTGGCTACGCTCAATCTGGCCTGTTGGCTGA<br>AATCACCCCGGACAAAGCGTTCCAGGACAAGCTGTATCCGTTTACCTGGGATGCCGTA<br>CGTTACAACGGCAAGCTGATTGCTTACCCGATCGCTGTTGAAGCGTTATCGCTGATTT<br>ATAACAAAGATCTGCTGCCGAACCCGCCAAAAACCTGGGAAGAGATCCCGGCGCTGGA<br>TAAAGAACTGAAAGCGAAAGGTAAGAGCGCGCTGATGTTCAACCTGCAAGAACCGTAC<br>TTCACCTGGCCGCTGATTGCTGCTGACGGGGGTTATGCGTTCAAGTATGAAAACGGCA<br>AGTACGACATTAAAGACGTGGGCGTGGATAACGCTGGCGCGAAAGCGGGTCTGACCTT	Construct encodes following protein: His$_{10}$-tag-linker-MBP-linker-TEV cleavage site-linker-Slr1599

CCTGGTTGACCTGATTAAAAACAAACACATGAATGCAGACACCGATTACTCCATCGCA<br>GAAGCTGCCTTTAATAAAGGCGAAACAGCGATGACCATCAACGGCCCGTGGGCATGGT<br>CCAACATCGACACCAGCAAAGTGAATTATGGTGTAACGGTACTGCCGACCTTCAAGGG<br>TCAACCATCCAAACCGTTCGTTGGCGTGCTGAGCGCAGGTATTAACGCCGCCAGTCCG<br>AACAAAGAGCTGGCAAAAGAGTTCCTCGAAAACTATCTGCTGACTGATGAAGGTCTGG<br>AAGCGGTTAATAAAGACAAACCGCTGGGTGCCGTAGCGCTGAAGTCTTACGAGGAAGA<br>GTTGGCGAAAGATCCACGTATTGCCGCCACCATGGAAAACGCCCAGAAAGGTGAAATC<br>ATGCCGAACATCCCGCAGATGTCCGCTTTCTGGTATGCCGTGCGTACTGCGGTGATCA<br>ACGCCGCCAGCGGTCGTCAGACTGTCGATGAAGCCCTGAAAGACGCGCAGACTgatta<br>cgatatcccaggtaccGAGAACCTGTACTTCCAATCCaatgccatgAATTGTCTCGCG<br>CTGTTTGACATCTCTTCCCCGTGGCCATGGTTAACTCCATCTCATGGTATTATTGCTG<br>CCTATCTGCTGTTGTTTGCGATTGTTCACTCGGGGCTGGCTGCCTTACGTGCGTGGGG<br>TGAATCCAAAATCGGTGCCCGTGGGTATCGTGTAATTTTTGCACTGGTGAGTATCCCA<br>TTAGCAACTGGTCTTATTATTTATTTCTTCAATCACCGTTATGACGGTTTACAGTTAT<br>GGCAAGTACAGGGCGTAACAGGGGTAAAGCCTTTAGTATGGATTCTCAGCGCTTTGAG<br>TTTTTTCTTCCTTTTCCCGGCGACGTTTAATCTGTTAGAGATCGCTGCAATCCAGAAG<br>CCAGAGATCCATCTTTACGAAACCGGTATTATTCGTATCTGCCGGCATCCGCAAATGG<br>TCGGGCAAGTCATCTGGTGCATTGCACATACGCTTTGGCTTGGCACTACGTTCACGCT<br>GGTAACGTCCCTCGGCCTCATTGCCCATCATTGCTTCGCAGTCTGGCACGGGGATCGG<br>CGGTGGCAACACAAATATGGCGAAGCCTTTCTCGCCGTAAAGCAACGCACGAGCATTA<br>TTCCTTTTCAAGCCATTTGGGAAGGCCGTCAGATCCTTGTTTGGCAAGAGTTTATTAA<br>GCCAGCGTATGTCGGCGTTATGGGTTTTATCGCCTTGTTATGGTGGGGGCATCCATGG<br>TTAATGGTCATGACATCACGGGTAAATTGGTAG	
**MBP-*Synechocystis* Z-ISO fusion version 2 (plus YhnF):**<br><br>ATGCATCACCACCATCATCATCACCATCATCATtcttctggtgtagatctgggtaccA<br>AAATCGAAGAAGGTAAACTGGTAATCTGGATTAACGGCGATAAAGGCTATAACGGTCT<br>CGCTGAAGTCGGTAAGAAATTCGAGAAAGATACCGGAATTAAAGTCACCGTTGAGCAT<br>CCGGATAAACTGGAAGAGAAATTCCCACAGGTTGCGGCAACTGGCGATGGCCCTGACA<br>TTATCTTCTGGGCACACGACCGCTTTGGTGGCTACGCTCAATCTGGCCTGTTGGCTGA<br>AATCACCCCGGACAAAGCGTTCCAGGACAAGCTGTATCCGTTTACCTGGGATGCCGTA<br>CGTTACAACGGCAAGCTGATTGCTTACCCGATCGCTGTTGAAGCGTTATCGCTGATTT<br>ATAACAAAGATCTGCTGCCGAACCCGCCAAAAACCTGGGAAGAGATCCCGGCGCTGGA<br>TAAAGAACTGAAAGCGAAAGGTAAGAGCGCGCTGATGTTCAACCTGCAAGAACCGTAC<br>TTCACCTGGCCGCTGATTGCTGCTGACGGGGGTTATGCGTTCAAGTATGAAAACGGCA<br>AGTACGACATTAAAGACGTGGGCGTGGATAACGCTGGCGCGAAAGCGGGTCTGACCTT<br>CCTGGTTGACCTGATTAAAAACAAACACATGAATGCAGACACCGATTACTCCATCGCA<br>GAAGCTGCCTTTAATAAAGGCGAAACAGCGATGACCATCAACGGCCCGTGGGCATGGT<br>CCAACATCGACACCAGCAAAGTGAATTATGGTGTAACGGTACTGCCGACCTTCAAGGG<br>TCAACCATCCAAACCGTTCGTTGGCGTGCTGAGCGCAGGTATTAACGCCGCCAGTCCG<br>AACAAAGAGCTGGCAAAAGAGTTCCTCGAAAACTATCTGCTGACTGATGAAGGTCTGG<br>AAGCGGTTAATAAAGACAAACCGCTGGGTGCCGTAGCGCTGAAGTCTTACGAGGAAGA<br>GTTGGCGAAAGATCCACGTATTGCCGCCACCATGGAAAACGCCCAGAAAGGTGAAATC<br>ATGCCGAACATCCCGCAGATGTCCGCTTTCTGGTATGCCGTGCGTACTGCGGTGATCA<br>ACGCCGCCAGCGGTCGTCAGACTGTCGATGAAGCCCTGAAAGACGCGCAGACTgatta<br>cgatatcccaggtaccGAGAACCTGTACTTCCAATCCaatgccatgATGTCCACGGAT<br>TTAAAATTTTCACTGGTAACAACGATTATCGTCCTCGGTTTGATCGTAGCCGTGGGTT<br>TGACTGCCGCGCTGCACAATTGTCTCGCGCTGTTTGACATCTCTTCCCCGTGGCCATG<br>GTTAACTCCATCTCATGGTATTATTGCTGCCTATCTGCTGTTGTTTGCGATTGTTCAC<br>TCGGGGCTGGCTGCCTTACGTGCGTGGGGTGAATCCAAAATCGGTGCCCGTGGGTATC<br>GTGTAATTTTTGCACTGGTGAGTATCCCATTAGCAACTGGTCTTATTATTTATTTCTT<br>CAATCACCGTTATGACGGTTTACAGTTATGGCAAGTACAGGGCGTAACAGGGGTAAAG<br>CCTTTAGTATGGATTCTCAGCGCTTTGAGTTTTTTCTTCCTTTTCCCGGCGACGTTTA<br>ATCTGTTAGAGATCGCTGCAATCCAGAAGCCAGAGATCCATCTTTACGAAACCGGTAT<br>TATTCGTATCTGCCGGCATCCGCAAATGGTCGGGCAAGTCATCTGGTGCATTGCACAT<br>ACGCTTTGGCTTGGCACTACGTTCACGCTGGTAACGTCCCTCGGCCTCATTGCCCATC<br>ATTGCTTCGCAGTCTGGCACGGGGATCGGCGGTGGCAACACAAATATGGCGAAGCCTT	Construct encodes following protein: His_{10}-tag-linker-MBP-linker-TEV cleavage site-linker-YhnF-Slr1599

TCTCGCCGTAAAGCAACGCACGAGCATTATTCCTTTTCAAGCCATTTGGGAAGGCCGT<br>CAGATCCTTGTTTGGCAAGAGTTTATTAAGCCAGCGTATGTCGGCGTTATGGGTTTTA<br>TCGCCTTGTTATGGTGGGGGCATCCATGGTTAATGGTCATGACATCACGGGTAAATTG<br>GTAG	
**MBP-*Synechocystis* Z-ISO fusion version 3 (pMAL-c5X):**<br><br>ATGAAAATCGAAGAAGGTAAACTGGTAATCTGGATTAACGGCGATAAAGGCTATAACG<br>GTCTCGCTGAAGTCGGTAAGAAATTCGAGAAAGATACCGGAATTAAAGTCACCGTTGA<br>GCATCCGGATAAACTGGAAGAGAAATTCCCACAGGTTGCGGCAACTGGCGATGGCCCT<br>GACATTATCTTCTGGGCACACGACCGCTTTGGTGGCTACGCTCAATCTGGCCTGTTGG<br>CTGAAATCACCCCGGACAAAGCGTTCCAGGACAAGCTGTATCCGTTTACCTGGGATGC<br>CGTACGTTACAACGGCAAGCTGATTGCTTACCCGATCGCTGTTGAAGCGTTATCGCTG<br>ATTTATAACAAAGATCTGCTGCCGAACCCGCCAAAAACCTGGGAAGAGATCCCGGCGC<br>TGGATAAAGAACTGAAAGCGAAAGGTAAGAGCGCGCTGATGTTCAACCTGCAAGAACC<br>GTACTTCACCTGGCCGCTGATTGCTGCTGACGGGGGTTATGCGTTCAAGTATGAAAAC<br>GGCAAGTACGACATTAAAGACGTGGGCGTGGATAACGCTGGCGCGAAAGCGGGTCTGA<br>CCTTCCTGGTTGACCTGATTAAAAACAAACACATGAATGCAGACACCGATTACTCCAT<br>CGCAGAAGCTGCCTTTAATAAAGGCGAAACAGCGATGACCATCAACGGCCCGTGGGCA<br>TGGTCCAACATCGACACCAGCAAAGTGAATTATGGTGTAACGGTACTGCCGACCTTCA<br>AGGGTCAACCATCCAAACCGTTCGTTGGCGTGCTGAGCGCAGGTATTAACGCCGCCAG<br>TCCGAACAAAGAGCTGGCAAAAGAGTTCCTCGAAAACTATCTGCTGACTGATGAAGGT<br>CTGGAAGCGGTTAATAAAGACAAACCGCTGGGTGCCGTAGCGCTGAAGTCTTACGAGG<br>AAGAGTTGGCGAAAGATCCACGTATTGCCGCCACCATGGAAAACGCCCAGAAAGGTGA<br>AATCATGCCGAACATCCCGCAGATGTCCGCTTTCTGGTATGCCGTGCGTACTGCGGTG<br>ATCAACGCCGCCAGCGGTCGTCAGACTGTCGATGAAGCCCTGAAAGACGCGCAGACTa<br>attcgagctcgaacaacaacaacaataacaataacaacaacctcgggATCGAGGGAAG<br>GatttcacatATGTTTGATATTTCTTCTCCTTGGCCCTGGTTGACCCCCAGCCATGGC<br>ATTATCGCCGCCTACCTTCTGTTGTTTGCCATCGTTCATAGTGGCTTAGCCGCCCTGC<br>GAGCCTGGGGGAAAGCAAAATTGGCGCTAGGGGCTATCGAGTTATATTTGCCCTGGT<br>GAGTATTCCCCTGGCTACGGGATTAATTATTTATTTTTTCAACCATCGCTATGATGGT<br>CTGCAACTCTGGCAAGTACAGGGAGTAACGGGAGTTAAACCCCTGGTGTGGATTTTGT<br>CTGCCCTATCTTTTTTCTTCCTTTTTCCCGCCACTTTTAACCTGTTGGAAATTGCCGC<br>TATCCAAAAACCGGAAATCCATCTTTATGAAACGGGCATTATCCGTATTTGTCGCCAT<br>CCTCAAATGGTCGGTCAAGTAATTTGGTGCATTGCCCACACCCTCTGGTTGGGAACCA<br>CTTTTACTCTGGTTACCAGCTTGGGATTAATTGCCCACCATTGCTTTGCAGTCTGGCA<br>TGGCGATCGCCGTTGGCAACACAAATATGGCGAGGCTTTTCTAGCAGTGAAACAAAGA<br>ACTTCCATCATTCCCTTTCAAGCGATCTGGGAGGGAAGGCAAATTCTAGTGTGGCAGG<br>AATTTATCAAACCAGCCTATGTGGGGGTAATGGGTTTCATTGCCCTACTATGGTGGGG<br>CCATCCCTGGTTGATGGTGATGACCTCCAGAGTTAATTGGTAG	Construct encodes following protein: MBP-linker-Factor Xa cleavage site-linker-Slr1599
**MBP-*Synechocystis* Z-ISO fusion version 4 (pMAL-p5X):**<br><br>ATGAAAATAAAAACAGGTGCACGCATCCTCGCATTATCCGCATTAACGACGATGATGT<br>TTTCCGCCTCGGCTCTCGCCAAAATCGAAGAAGGTAAACTGGTAATCTGGATTAACGG<br>CGATAAAGGCTATAACGGTCTCGCTGAAGTCGGTAAGAAATTCGAGAAAGATACCGGA<br>ATTAAAGTCACCGTTGAGCATCCGGATAAACTGGAAGAGAAATTCCCACAGGTTGCGG<br>CAACTGGCGATGGCCCTGACATTATCTTCTGGGCACACGACCGCTTTGGTGGCTACGC<br>TCAATCTGGCCTGTTGGCTGAAATCACCCCGGACAAAGCGTTCCAGGACAAGCTGTAT<br>CCGTTTACCTGGGATGCCGTACGTTACAACGGCAAGCTGATTGCTTACCCGATCGCTG<br>TTGAAGCGTTATCGCTGATTTATAACAAAGATCTGCTGCCGAACCCGCCAAAAACCTG<br>GGAAGAGATCCCGGCGCTGGATAAAGAACTGAAAGCGAAAGGTAAGAGCGCGCTGATG<br>TTCAACCTGCAAGAACCGTACTTCACCTGGCCGCTGATTGCTGCTGACGGGGGTTATG<br>CGTTCAAGTATGAAAACGGCAAGTACGACATTAAAGACGTGGGCGTGGATAACGCTGG<br>CGCGAAAGCGGGTCTGACCTTCCTGGTTGACCTGATTAAAAACAAACACATGAATGCA<br>GACACCGATTACTCCATCGCAGAAGCTGCCTTTAATAAAGGCGAAACAGCGATGACCA<br>TCAACGGCCCGTGGGCATGGTCCAACATCGACACCAGCAAAGTGAATTATGGTGTAAC<br>GGTACTGCCGACCTTCAAGGGTCAACCATCCAAACCGTTCGTTGGCGTGCTGAGCGCA<br>GGTATTAACGCCGCCAGTCCGAACAAAGAGCTGGCAAAAGAGTTCCTCGAAAACTATC	Constructs encodes following protein: MalE signal sequence-MBP-linker-Factor Xa cleavage site-linker-Slr1599

TGCTGACTGATGAAGGTCTGGAAGCGGTTAATAAAGACAAACCGCTGGGTGCCGTAGC<br>GCTGAAGTCTTACGAGGAAGAGTTGGCGAAAGATCCACGTATTGCCGCCACCATGGAA<br>AACGCCCAGAAAGGTGAAATCATGCCGAACATCCCGCAGATGTCCGCTTTCTGGTATG<br>CCGTGCGTACTGCGGTGATCAACGCCGCCAGCGGTCGTCAGACTGTCGATGAAGCCCT<br>GAAAGACGCGCAGACTaattcgagctcgaacaacaacaacaataacaataacaacaac<br>ctcgggATCGAGGGAAGGatttcacatATGTTTGATATTTCTTCTCCTTGGCCCTGGT<br>TGACCCCCAGCCATGGCATTATCGCCGCCTACCTTCTGTTGTTTGCCATCGTTCATAG<br>TGGCTTAGCCGCCCTGCGAGCCTGGGGGGAAAGCAAAATTGGCGCTAGGGGCTATCGA<br>GTTATATTTGCCCTGGTGAGTATTCCCCTGGCTACGGGATTAATTATTTATTTTTTCA<br>ACCATCGCTATGATGGTCTGCAACTCTGGCAAGTACAGGGAGTAACGGGAGTTAAACC<br>CCTGGTGTGGATTTTGTCTGCCCTATCTTTTTTCTTCCTTTTTCCCGCCACTTTTAAC<br>CTGTTGGAAATTGCCGCTATCCAAAAACCGGAAATCCATCTTTATGAAACGGGCATTA<br>TCCGTATTTGTCGCCATCCTCAAATGGTCGGTCAAGTAATTTGGTGCATTGCCCACAC<br>CCTCTGGTTGGGAACCACTTTTACTCTGGTTACCAGCTTGGGATTAATTGCCCACCAT<br>TGCTTTGCAGTCTGGCATGGCGATCGCCGTTGGCAACACAAATATGGCGAGGCTTTTC<br>TAGCAGTGAAACAAAGAACTTCCATCATTCCCTTTCAAGCGATCTGGGAGGGAAGGCA<br>AATTCTAGTGTGGCAGGAATTTATCAAACCAGCCTATGTGGGGGTAATGGGTTTCATT<br>GCCCTACTATGGTGGGGCCATCCCTGGTTGATGGTGATGACCTCCAGAGTTAATTGGT<br>AG	

Table S4. ROSETTA membrane protein scores for abolishment of putative H150 binding site in *Zea mays* Z-ISO.

Mutation	ROSETTA score	Mutation	ROSETTA score
WT	-760.705	Arg296Trp	-663.212
Arg170Ala	-762.904	Arg296Phe	-732.487
Arg170Trp	-755.944	Gln255Ala	-766.112
Arg170Phe	-642.435	Gln255Trp	-756.624
Arg296Ala	-758.187	Gln255Phe	-759.713

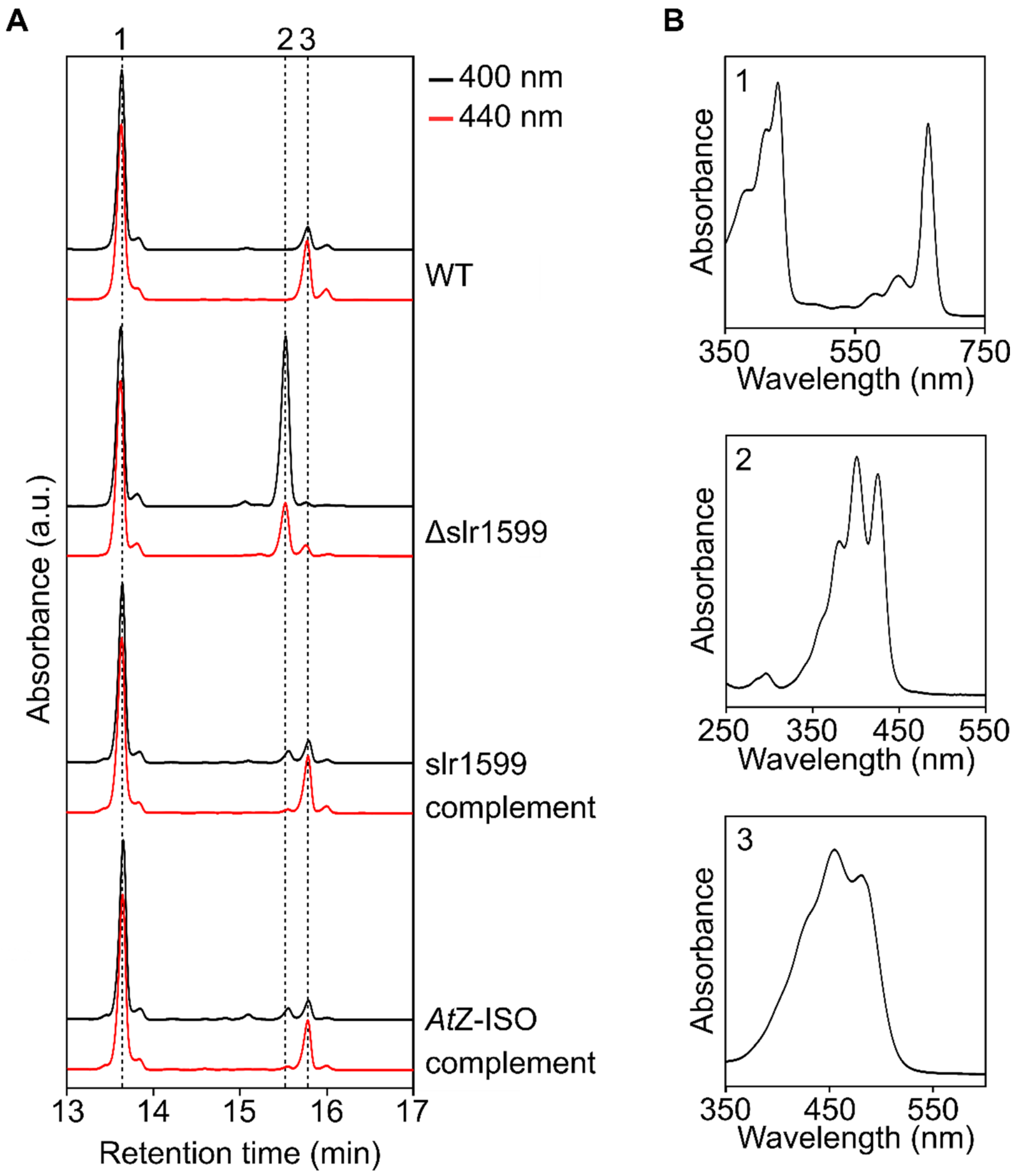

Figure S1. Complementation of the *Synechocystis* Δslr1599 mutant. (A) Pigments were extracted from WT, Δslr1599 and Δslr1599 with slr1599 or the *Arabidopsis* Z-ISO (*At*Z-ISO) genes inserted into the *psbAII* locus (slr1599 complement and *At*Z-ISO complement, respectively) and separated by RP-HPLC. Eluted pigments were detected by monitoring absorbance at 400 nm (black trace) and 440 nm (red trace). Peak heights were normalized to the Chl peak (peak 1). Dotted lines and numbers correspond to numbers in (B). **(B)** Eluted pigments in (A) were identified by their absorbance spectra. Numbers correspond to equivalent numbered peaks highlighted by dotted lines in (A). Peak 1 = chl *a*; peak 2 = 9,15,9′-tri-*cis*-ζ-carotene; peak 3 = β-carotene.

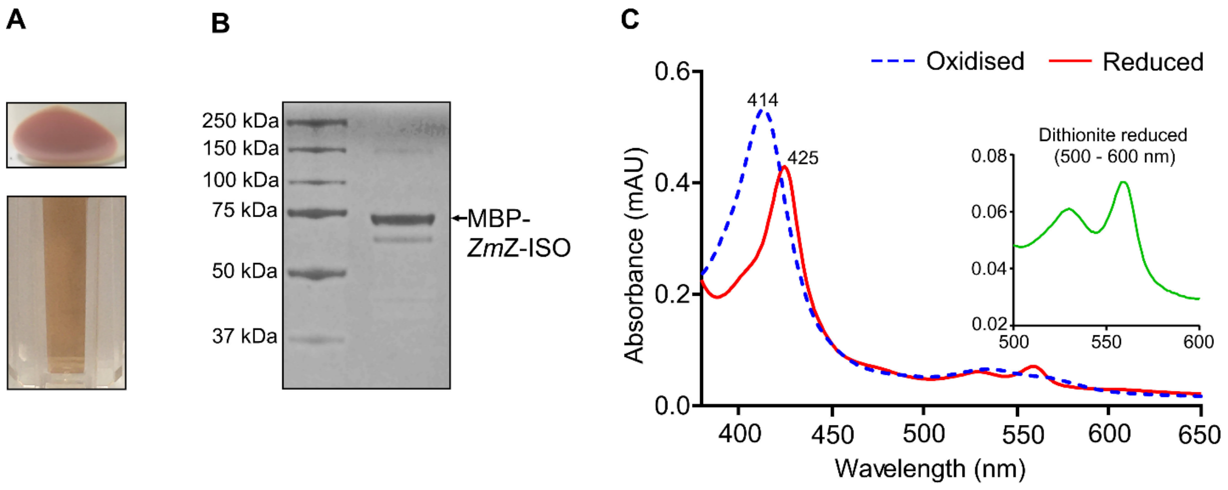

Figure S2. Purification of the *Zea mays* (*Zm*) MBP-Z-ISO fusion protein. (A) Cell pellets (top) and solubilized membrane fractions (bottom) of MBP-*Zm*Z-ISO heterologously produced in *E. coli*. **(B)** SDS-PAGE analysis of purified MBP-*Zm*Z-ISO. **(C)** Oxidized and reduced spectra of the MBP-*Zm*Z-ISO protein with spectral changes in the 500-600 nm region following sodium dithionite reduction highlighted in the inset panel.

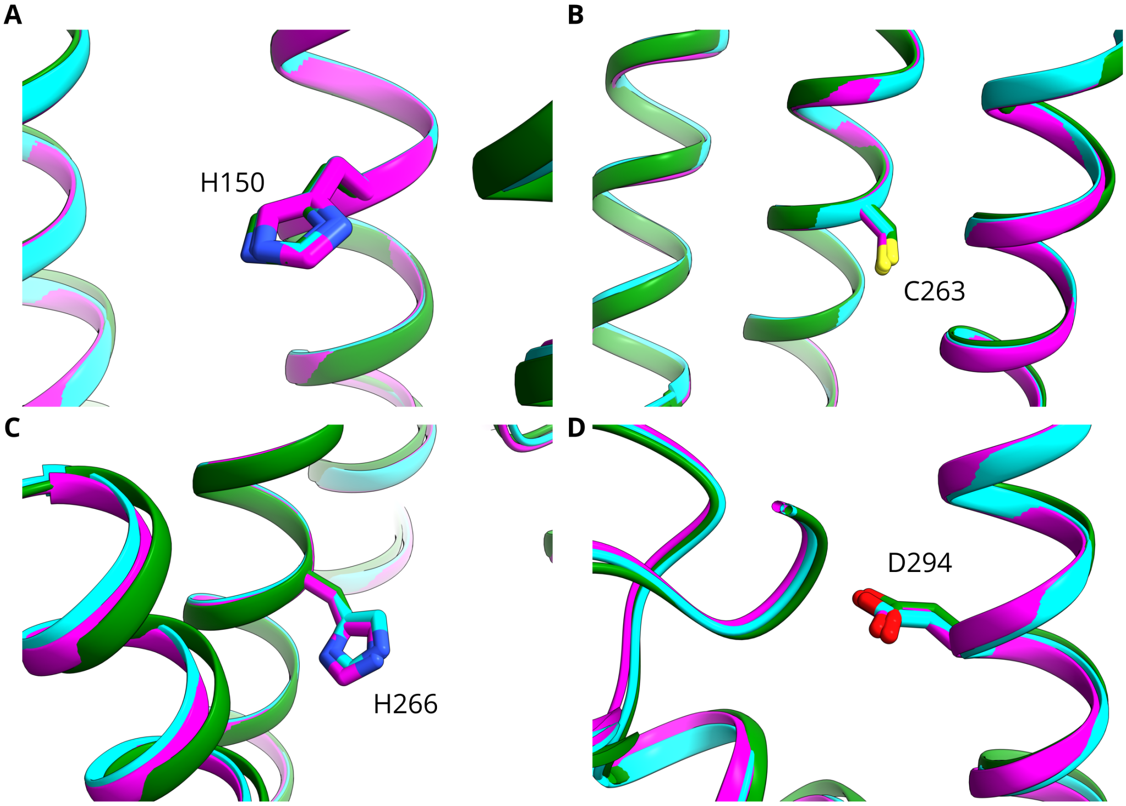

Figure S3. Orientation of conserved Z-ISO residues with putative catalytic activity. Z-ISO model structures from *Synechocystis* (green), *Arabidopsis* (cyan) and maize (magenta) are shown, with atomic coordinates of previously-proposed heme binding residues – His 150 (A) and His 266 (C) – and residues with catalytic activity – Cys 263 (B) and Asp 294 (D) – displayed as sticks (oxygen, red; nitrogen, blue; sulfur, yellow).

A

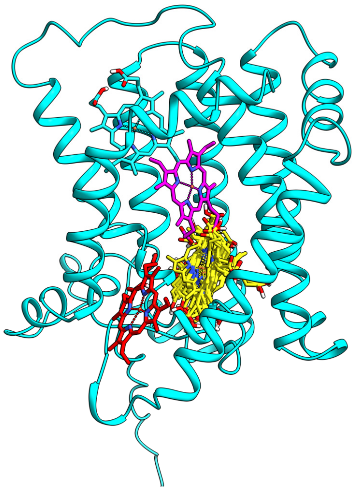

Binding mode	Binding energy (kcal/mol)
1	-9.7
2	-9.2
3	-9.1
4	-9.0
5	-9.0
6	-8.9
7	-8.9
8	-8.8
9	-8.8
10	-8.7
11	-8.7
12	-8.6
13	-8.5
14	-8.4
15	-8.3
16	-8.3
17	-8.2
18	-8.1
19	-8.0
20	-7.9

B

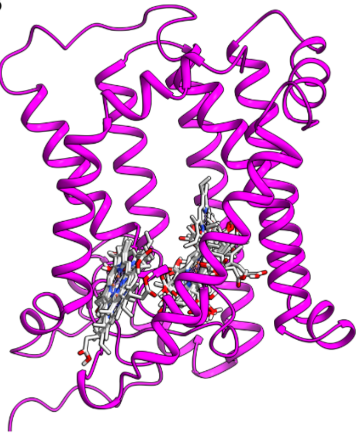

Binding mode	Binding energy (kcal/mol)
1	-9.7
2	-9.3
3	-9.2
4	-9.2
5	-9.1
6	-9.1
7	-9.0
8	-9.0
9	-8.9
10	-8.8
11	-8.8
12	-8.8
13	-8.8
14	-8.7
15	-8.6
16	-8.5
17	-8.3
18	-8.2
19	-8.1
20	-8.1

Figure S4. Heme B docking in *Arabidopsis* (A) and maize (B) Z-ISO using Autodock Vina. Model protein structures (ribbon) are shown with the heme B cofactor (sticks) positioned in all 20 geometries identified in the docking simulation. The color of the cofactor in (A) corresponds to the color of the cell for the tabulated predicted binding energy estimate displayed in the inset table.

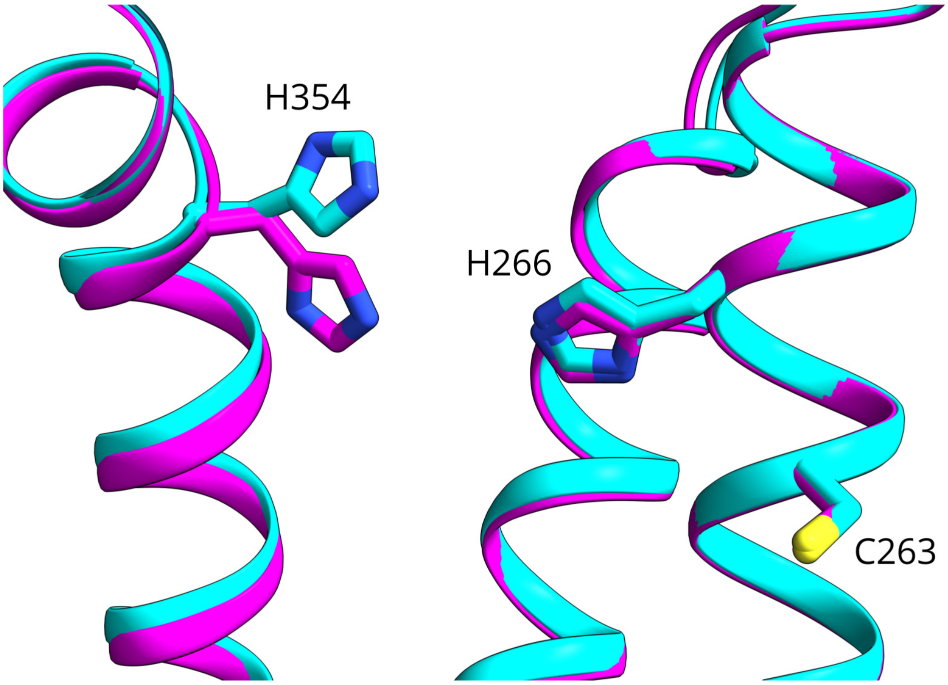

Figure S5. Alignment of H266 and H354, relative to C263, in maize (magenta) and *Arabidopsis* (cyan) Z-ISO. Key residues displayed as sticks (nitrogen, blue; sulfur, yellow).

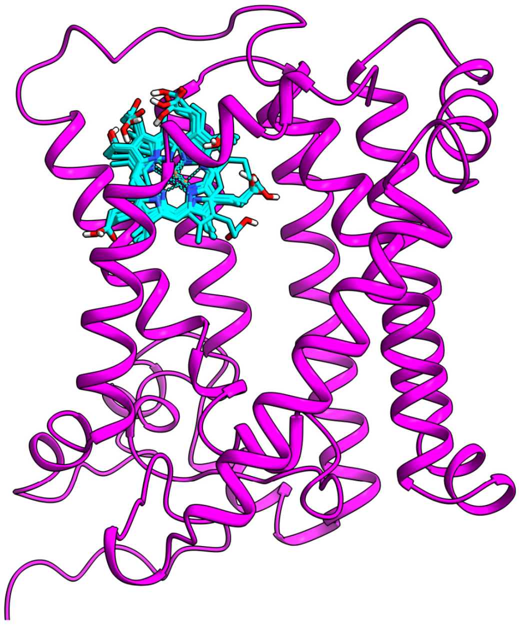

Binding mode	Binding energy (kcal/mol)
1	-4.7
2	-3.6
3	-3.6
4	-3.5
5	-3.4
6	-3.3
7	-2.0
8	-2.0

Figure S6. Heme docking at the H266/H354 site in maize Z-ISO. Corresponding predicted binding energy values for each heme binding mode are displayed in the table inset (protein, purple; heme-carbon, cyan; oxygen, red; nitrogen, blue; hydrogen, white).

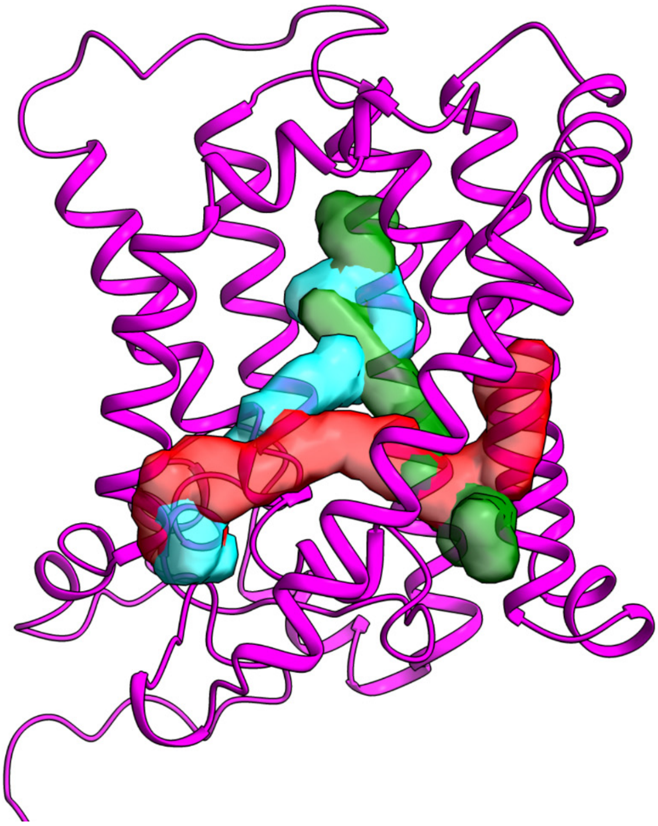

Binding mode	Binding energy (kcal/mol)
1	-9.4
2	-9.4
3	-9.3

Figure S7. Docking 9,15,9'-tri-*cis*-ζ-carotene into the maize Z-ISO-heme B model. The corresponding predicted binding energy values for each carotenoid binding mode are displayed in the inset table. The color of the carotenoid density corresponds to the color of the cell in the table.

A

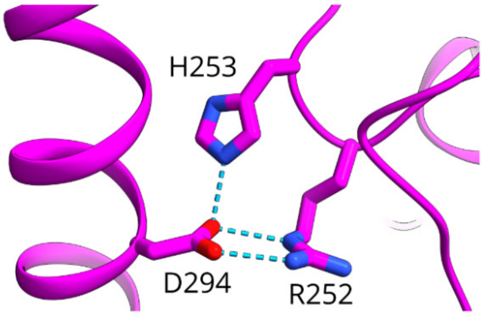

Mutant	Rosetta score (REU)
WT	-1108.670
R252A	-1097.550
H253A	-1104.181
D294A	-1099.856

B

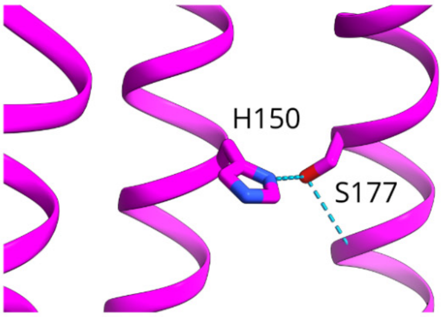

Mutant	Rosetta score (REU)
WT	-1108.670
S177A	-1105.497
H150A	-1104.429

C

Mutant	Rosetta Score (REU)
WT	-1108.670
R170A	-1105.290
R170F	-968.907
R170W	-1094.132
Q255A	-1105.130
Q255F	-1102.610
Q255W	-1085.195
R296A	-1101.248
R296F	-715.522
R296W	-892.455
H354A	-1107.523
H354W	-1103.022

Figure S8. Using the Rosetta score function to estimate free-energy changes of point mutations. Predicted hydrogen-bonds between residues proximal to D294 (A) and H150 (B) are shown as dashed lines. Note that only side chain residues are rendered as sticks, backbone hydrogen-bond donors are not visualized. Estimated free-energy changes for point mutations around residues H253 (A, C), H150 (B, C) are displayed in the tables in Rosetta energy units (REU).
